# Spontaneous mutations that confer resistance to 2-deoxyglucose act through Hxk2 and Snf1 pathways to regulate gene expression and HXT endocytosis

**DOI:** 10.1371/journal.pgen.1008484

**Published:** 2020-07-16

**Authors:** Samantha R. Soncini, Dakshayini G. Chandrashekarappa, David A. Augustine, Kenny P. Callahan, Allyson F. O’Donnell, Martin C. Schmidt

**Affiliations:** 1 Department of Microbiology and Molecular Genetics, University of Pittsburgh School of Medicine, Pittsburgh, Pennsylvania, United States of America; 2 Department of Biological Sciences, University of Pittsburgh, Pittsburgh, Pennsylvania, United States of America; 3 Marlboro College, Marlboro Vermont, United States of America; Pacific Northwest Research Institute, UNITED STATES

## Abstract

Yeast and fast-growing human tumor cells share metabolic similarities in that both cells use fermentation of glucose for energy and both are highly sensitive to the glucose analog 2-deoxyglucose. Spontaneous mutations in *S*. *cerevisiae* that conferred resistance to 2-deoxyglucose were identified by whole genome sequencing. Missense alleles of the *HXK2*, *REG1*, *GLC7* and *SNF1* genes were shown to confer significant resistance to 2-deoxyglucose and all had the potential to alter the activity and or target selection of the Snf1 kinase signaling pathway. All three missense alleles in *HXK2* resulted in significantly reduced catalytic activity. Addition of 2DG promotes endocytosis of the glucose transporter Hxt3. All but one of the 2DG-resistant strains reduced the 2DG-mediated hexose transporter endocytosis by increasing plasma membrane occupancy of the Hxt3 protein. Increased expression of the DOG (deoxyglucose) phosphatases has been associated with resistance to 2-deoxyglucose. Expression of both the *DOG1* and *DOG2* mRNA was elevated after treatment with 2-deoxyglucose but induction of these genes is not associated with 2DG-resistance. RNAseq analysis of the transcriptional response to 2DG showed large scale, genome-wide changes in mRNA abundance that were greatly reduced in the 2DG resistant strains. These findings suggest the common adaptive response to 2DG is to limit the magnitude of the response. Genetic studies of 2DG resistance using the dominant *SNF1-G53R* allele in cells that are genetically compromised in both the endocytosis and *DOG* pathways suggest that at least one more mechanism for conferring resistance to this glucose analog remains to be discovered.

## Author summary

Yeast and fast-growing human tumor cells share metabolic similarities in that both cells use fermentation of glucose for energy and both are highly sensitive to the glucose analog 2-deoxyglucose. Another similarity between yeast cells and human tumor cells is that both cells can acquire resistance to 2-deoxyglucose, an outcome that can limit the usefulness of some cancer therapeutics. In this study, we used bakers’ yeast as a model organism to better understand the mechanism of toxicity and acquisition of resistance to 2-deoxyglucose. Spontaneous mutations in *S*. *cerevisiae* that conferred resistance to 2-deoxyglucose were isolated and identified by whole genome sequencing, a technology that was not available until recently. Our studies indicate that 2-deoxyglucose becomes toxic after it is phosphorylated by an enzyme called hexokinase. One important route to resistance is to reduce hexokinase activity. Other parallel pathways to resistance include increased expression of a hydrolase that degrades the toxic metabolite, altered localization of glucose transporters and altered glucose signal transduction pathways.

## Introduction

Carbon source acquisition is necessary for life and the preferred carbon source for the vast majority of cells is glucose. *Saccharomyces cerevisiae* cells dedicate considerable regulatory effort coordinating the expression and localization of glucose transporters to optimize glucose uptake under a wide range of glucose concentrations. However, unlike most cells, yeast ferment glucose to ethanol even in the presence of oxygen rather than utilizing aerobic respiration. Similarly, most human cancer cells shun aerobic respiration in favor of fermentation of glucose to lactate in a process known as the Warburg effect [1]. These metabolic similarities and the shared reliance on efficient glucose uptake make yeast a good model system for understanding the metabolic rewiring that allows rapid proliferation in cancer cells.

The ‘addiction’ of cancer cells to glucose uptake is well documented and makes cancer cells particularly susceptible to a toxic analog of glucose, known as 2-deoxyglucose (2DG). Understanding the mechanism of action of 2DG is clinically important as this drug is in active clinical trials as an anti-cancer therapeutic [2, 3]. As with any cancer therapeutic, it is critical to understand the mechanism(s) by which cancerous cells evade pharmacological intervention and become resistant to treatment. In this work, we aim to discover the mechanism(s) of 2DG resistance using a screen for spontaneous suppressors of 2DG toxicity in yeast. By defining the pathways that lead to 2DG-resistance, we may better be able to design combinatorial treatments for cancer cells that spontaneously mutate to evade 2DG-induced toxicity.

Genetic selection for mutations in yeast that confer resistance to 2DG have been undertaken since the 1970s [4], and these studies have been instrumental in defining the genes that regulate glucose repression [4–7]. However, all of the prior 2DG screens were conducted on media that contained alternative carbon sources (e.g., sucrose, raffinose, galactose), rather than on the preferred carbon source of glucose. Mutations that relieved glucose repression of gene expression were isolated, including mutations in the *REG1*, *HXK2* and *GRR1* genes. These findings led to the hypothesis that 2DG promoted glucose repression, thus inhibiting growth on alternative carbon sources. However, recent studies with 2DG using glucose as the carbon source have challenged this hypothesis [8, 9], since promotion of glucose repression should not be deleterious to cells growing on glucose. We defined a new mechanism for 2DG-induced toxicity in yeast that hinges upon the ability of the AMP-activated protein kinase (AMPK) to regulate the membrane trafficking of the high-capacity glucose transporters, Hxt1 and Hxt3 [10]. In the absence of Snf1 (the yeast ortholog of mammalian AMPK), cells become hypersensitive to 2DG [8] and have greatly decreased plasma membrane retention of Hxt1 and Hxt3 [10]. Reduced Hxt1 and Hxt3 abundance at the plasma membrane is due to increased endocytic turnover of these transporters mediated by the α-arrestins Rod1 and Rog3, both of which are phosphorylated and inhibited by the Snf1 kinase [10, 11]. An analogous signaling pathway has been identified in mammalian cells, where 2DG promotes AMPK-mediated phosphorylation of the α-arrestin TXNIP and impedes TXNIP-mediated endocytosis of the glucose transporter GLUT1 [12]. While identifying this conserved pathway represents a leap forward in our understanding of 2DG-induced rewiring in the cell, it remained unclear what other pathways might also be important drivers of 2DG-resistance.

An alternative genetic screen for 2DG-resistance using an overexpression strategy identified the 2-deoxyglucose-6-phosphatate phosphatase (2DG-6P) genes, *DOG1* and *DOG2* [13]. These genes encode phosphatases in the HAD-family (halo-acid dehalogenase) most closely related to the glycerol-3-phosphate phosphatases (*GPP1* and *GPP2*) with the signature catalytic motif DxDxT [14] present at their N-termini. It has been proposed that these phosphatases confer resistance to 2DG by dephosphorylating the 2DG-6P produced when hexose kinases act on cellular 2DG; 2DG-6P is thought to be a toxic metabolite that accumulates within the cell after treatment with 2DG, however metabolomics studies of 2DG-treated cells are needed to confirm this idea. A recent study aimed at defining the mechanism of 2DG resistance using an unbiased proteomics approach found that increased DOG phosphatase abundance induced 2DG resistance in yeast [15]. Multiple signaling pathways including the Hog1 map kinase pathway, the unfolded protein response pathway and the cell wall integrity pathway, mediated this 2DG-induced upregulation of the *DOG1* and *DOG2* genes. Furthermore, overexpression of a related human HAD phosphatase could impart 2DG resistance in a human cell line, suggesting that this mechanism of resistance is conserved between yeast and humans.

In this study, we undertook a new selection for 2DG-resistant yeast strains using novel approaches in an effort to map the mechanism by which cells could spontaneously acquire 2DG-resistance. In contrast to earlier studies, we selected for spontaneous 2DG-resistant strains by growing on glucose rather than alternative carbon sources. Thus, the initial metabolic program would more like the high glucose environment a typical cancerous cell would experience. Second, we included an integrated *HXT3-GFP* reporter gene in our starting strain in order to determine if 2DG-resistant mutants caused changes in membrane trafficking of this glucose transporter. Finally, we used whole genome sequencing to identify mutations in our resistant strains in an unbiased way. Using this approach, we defined new recessive alleles in *HXK2*, *GLC7* and *REG1* that confer 2DG resistance. We also recovered a dominant allele of *SNF1* that had previously been isolated in a screen for Snf1 kinase activity in the absence of the kinase’s gamma subunit [16]. We further demonstrate that mutations in *HXK2* regulate endocytosis of Hxt3, marking the first time Hxk2 has been shown to alter protein trafficking events. Using RNAseq analyses, we define the transcriptional response to 2DG in wild type cells and in our 2DG-resistant mutants. Wild type cells respond to 2DG by making large scale changes in their transcription profile and in particular, by downregulating the transcription of mRNAs encoding ribosomal proteins. Our 2DG-resistant cells all share a common adaptive response to 2DG. They exhibit a muted response as seen by reducing the 2DG-mediated endocytosis of Hxt3 and by greatly reducing the magnitude of the transcriptional response. These finding suggest that the most adaptive response to 2DG is to limit the magnitude of the response. Importantly, while 2DG treatment induces expression of the *DOG1* and *DOG2* mRNAs, our 2DG-resistant mutants show reduced, not elevated DOG expression compared to wild type cells. Finally, our genetic analyses suggest the existence of at least one additional yet-to-be-defined pathway that can confer 2DG resistance.

## Results

### Isolation and identification of spontaneous 2DG-resistant mutants

In order to obtain new, independent, and spontaneous 2DG-resistant mutants in *S*. *cerevisiae*, single colonies of wild-type yeast cells were grown overnight in synthetic complete media with 2% glucose as the carbon source. Cultures were then diluted in water, and 2x10^7^ cells were spread onto agar plates containing synthetic complete media with 2% glucose and 0.1% 2DG. Plates were incubated for 4–6 days until single colonies grew and could be streaked onto a fresh plate without 2DG. Isolation of 2DG-resistant strains in this way generated 6–10 colonies per plate. Single colonies from these initial isolates were then tested for growth on media with and without 2DG. Since we showed previously that deletion of the *HXK2* and *REG1* genes conferred 2DG resistance to cells using glucose as the carbon source [8], we expected to recover loss of function alleles in these genes. Haploid strains were mated to *hxk2Δ* and *reg1Δ* strains, and the resulting diploids were scored for complementation. Twenty-eight 2DG-resistant haploid strains were examined. Of these, 21 were in the same complementation group as *hxk2Δ*, 1 was in the same complementation group as *reg1Δ*, and 6 were not in either of these complementation groups. Mating these 2DG-resistant strains with a wild type strain showed that 27 of the mutants contained recessive alleles conferring 2DG resistance and one strain contained a dominant allele.

In order to identify the mutations that confer 2DG resistance, we isolated genomic DNA from the 2DG-resistant strains and subjected it to whole genome sequencing using Illumina NextSeq500. This produced 151-bp paired-end reads [17] with an average depth of 20 million reads per sample. At this depth, each nucleotide in the yeast genome was sequenced an average of 240 times. We identified candidate mutations in 19 of the 22 haploid strains sequenced (Table 1). Of the many strains in the *hxk2Δ* complementation group, 15 were subjected to whole genome sequencing. Of these, 12 had the same mutation producing the missense allele, *hxk2-G55V*. We also isolated *hxk2-D417G* twice and *hxk2-R423T* once. We identified candidate mutations in four haploid strains that produced missense alleles in the *SNF1*, *REG1*, and *GLC7* genes. The one resistant strain with a dominant mutation contained the missense allele *SNF1-G53R*. This same allele had been isolated previously in a screen for Snf1 kinase function in the absence of the complex’s gamma subunit, Snf4 [16]. Three recessive alleles produced missense mutations in the yeast protein phosphatase I complex comprised of Glc7 and Reg1 [14]. We isolated *glc7-Q48K* twice and *reg1-P231L* once. Three strains that were 2DG resistant but lacked candidate mutations were found to be aneuploid with extra copies of multiple chromosomes (S1 Fig). Further characterization of the aneuploid strains will be the focus of future work.

**Table 1:** Genomic sequencing results.

Isolate name	Inheritance	Candidate Mutations Detected
RS5, RS8	Recessive	HXK2-D417G
RS6, RS7, RS15, RS16, RS21, RS22, RS23, RS27, RS29, RS30, RS31, RS32	Recessive	HXK2-G55V
RS20	Recessive	HXK2-R423T
RS25, RS28	Recessive	GLC7-Q48K
RS26	Recessive	REG1-P231L
RS24	Dominant	SNF1-G53R
RS17	Intermediate	Haploid with disomy of chromosomes 3, 8 and 13
RS18	Intermediate	Haploid with disomy of chromosomes 3, 8, 11 and 13
RS19	Intermediate	Haploid with disomy of chromosomes 3, 8, 11 and 13

In order to confirm that these candidate mutations in the *SNF1*, *HXK2*, *GLC7* and *REG1* genes did in fact cause 2DG resistance, the 2DG-resistant strains were all mated to a wild type 2DG-sensitive strain and subjected to sporulation. The haploid progeny all showed 2:2 segregation of 2DG-resistance demonstrating that a single mutation was responsible for the 2DG-resistance phenotype. We engineered plasmids to contain these mutations and introduced these plasmids into strains lacking the cognate gene. In all cases, the candidate mutations when present on low-copy plasmids produced comparable 2DG resistance as was observed when the mutations were in the chromosomes of the original isolate (S2 Fig). This finding rules out the possibility that additional variants present in the original isolates were contributing significant resistance to 2DG. We concluded that these single mutations were sufficient to confer the 2DG-resistant phenotypes. We next sought to characterize each mutation further and uncover how they play a role in in 2DG resistance.

### Recessive, missense alleles in *HXK2* gene inhibit catalytic activity and confer 2DG resistance

We used oligonucleotide-directed mutagenesis to generate several versions of *HXK2* all with a C-terminal epitope tag. We made the three missense alleles isolated in this screen (*hxk2*-G55V, *hxk2*-D417G, and *hxk2*-R423T). We also tagged two additional alleles of *HXK2* that had been previously reported to separate the catalytic and gene regulatory functions of Hxk2 [18]. These alleles are referred to as *HXK2*-wca (without catalytic activity) and *HXK2*-wrf (without regulatory function). We previously reported that the *HXK2-wca* mutation conferred 2DG-resistance, while the *HXK2-wrf* mutation did not confer 2DG resistance [8]. Finally, we created the *hxk2*-D211A allele, since the aspartate at 211 is a catalytic residue and the substitution of this residue with alanine is known to severely compromise catalytic activity [19].

**Fig 1:**
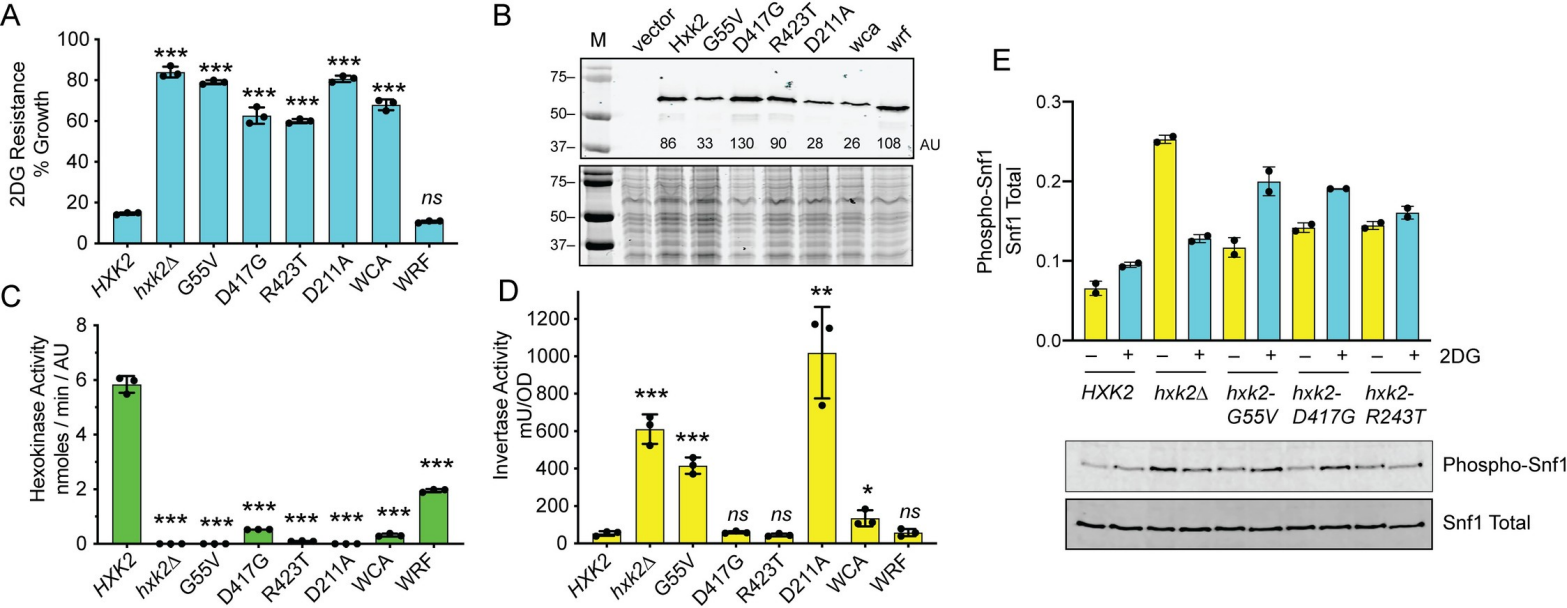
Amino acid substitutions in Hxk2 confer resistance to 2DG. **A.** 2DG-resistance assay in *hxk2Δ* cells transformed with low-copy plasmids encoding wild type (WT) *HXK2*, *HXK2* with the indicated amino acid substitutions, the wca and wrf alleles [18] or empty vector (*hxk2Δ*). Three independent cultures were grown in 2% glucose in media with and without 0.1% 2DG with percent growth plotted relative to growth in the absence of 2DG. Individual data points are shown with the mean ± SD indicated with a solid bar. Values statistically different from wild type are indicated. **B.** Western blot of Hxk2 proteins tagged with three copies of the V5 epitope at the C-terminus. Cells were grown in synthetic complete medium with 2% galactose. Quantitation of the western signal in arbitrary units (AU) is shown. As a control for equal loading, Coomassie stained gel of the same extracts is shown below. **C.** Catalytic activity of hexokinase was measured in protein extracts of cells lacking all three hexokinase genes (*hxk1Δ hxk2Δ glk1Δ*). Cells were transformed with the same low-copy plasmids in A and grown as in B. Activity is expressed in nmoles per minute normalized to the amount of protein in the extract as determined by western blotting. Values statistically different from wild type are indicated. **D.** Invertase activity measured in three independent transformants of *hxk2Δ* cells grown in media with 2% glucose and with the same plasmids used in A. Values statistically different from wild type are indicated. **E.** Snf1 kinase activation was measured in duplicate cultures before and after treatment with 2DG. Ratio of phosphorylated Snf1 over total Snf1 is plotted with representative western blots shown below.

The three *HXK2* alleles isolated in this screen were found to be highly 2DG resistant (Fig 1A). Complete deletion of the *HXK2* gene conferred the greatest resistance to 2DG with the *hxk2*-G55V and *hxk2*-D211A alleles following close behind. Consistent with our earlier studies [8], the *HXK2-wca* allele conferred 2DG resistance while the *HXK2-*wrf allele did not. To assess Hxk2 protein expression and catalytic activity, we utilized a strain lacking all three hexokinase genes (*hxk1Δ hxk2Δ glk1Δ*). This strain can grow on galactose but is unable to grow by fermentation of glucose (S3A Fig). The triple hexokinase delete strain was transformed with low-copy plasmids expressing epitope-tagged wild type or one of the six alleles of *HXK2* described above. A western blot of protein extracts from cells grown on galactose demonstrates that all proteins were expressed as full-length proteins although the absolute level of Hxk2 protein accumulation showed some variation (Fig 1B).

Hexokinase II is reported to have two distinct activities, a catalytic activity and a gene regulatory activity [18, 20]. We measured both of these activities in the Hxk2 mutants under study. First, we used a coupled *in vitro* enzyme assay system [21, 22] to measure the catalytic activity of each Hxk2 enzyme (Fig 1C). Catalytic rate (nmoles/min) was normalized to the amount of Hxk2 protein as quantified by western blotting (Fig 1B) using the arbitrary units (au) shown. Extracts from cells lacking any hexokinase and from cells expressing the D211A allele did not contain any detectable hexokinase activity. All the mutant Hxk2 enzymes isolated in this study showed severely compromised catalytic activity ranging from 3% to 0.3% of that observed in cells expressing wild type Hxk2. The catalytic activity and the 2DG resistance phenotype show a very strong inverse correlation. The mutant enzyme with the most catalytic activity (hxk2-wrf) confers the least 2DG resistance, while the mutants with the least catalytic activity (hxk2-G55V and hxk2-D211A) showed the highest levels of 2DG resistance. One curious observation about the three yeast hexokinase isozymes (*HXK1*, *HXK2* and *GLK1*) is that only deletion of *HXK2* confers 2DG resistance (S3A Fig). This observation could be explained if the Hxk2 enzyme was the only one of these isozymes capable of phosphorylating 2DG. We tested this in our *in vitro* assay and found that both Hxk1 and Hxk2 were able to phosphorylate 2DG *in vitro*. In fact, the Hxk1 enzyme had a higher affinity (lower Km) and higher Vmax for 2DG than the Hxk2 enzyme (Table 2 and S4 and S5 Figs). The Glk1 enzyme exhibited a much higher affinity (lower Km) for glucose than either Hxk1 or Hxk2 but was not able to detectably phosphorylate 2DG in our *in vitro* assay (S6 Fig). We suspect that Glk1 is able to phosphorylate 2DG to some extent *in vivo* since introduction of *GLK1* to the triple hexokinase mutant confers sensitivity to 2DG when growing on glycerol/ethanol media (S3B Fig).

**Table 2:** Kinetic analysis of hexokinases.

Hexokinase	Km Glucose (mM)	Vmax Glucose (nmole/min)	Km 2DG (mM)	Vmax 2DG (nmole/min)
*HXK1*	0.11	0.42	0.12	0.26
*HXK2*	0.19	0.26	0.28	0.075
*GLK1*	0.055	0.17	ND^a^	ND^a^

^a^ND, not detected

In order to assess the gene regulatory function of Hxk2, we measured invertase expression in cells grown on high glucose. Previous studies have shown that invertase expression is repressed in wild-type cells growing on high glucose but is significantly derepressed (induced) when the *HXK2* gene has been deleted or mutated [5, 20, 23]. Invertase expression is a complicated, multistep process involving changes in transcription, protein translation, modification and secretion. However, invertase activity has been used as a readout for *HXK2* gene function in numerous papers over several decades and provides means to compare alleles isolated in different studies. We found that the cells containing the lowest levels of Hxk2 catalytic activity (*hxk2Δ*, *hxk2-G55V* and *hxk2-D211A*) had the greatest derepression of invertase (Fig 1D). Surprisingly, some *HXK2* alleles (*hxk2-D417G*, *hxk2-R423T* and *hxk2-wca*) showed normal levels of invertase repression even though they were all severely compromised for hexokinase catalytic activity (Fig 1B). This observation is consistent with a previous study finding little correlation between catalytic activity and invertase repression [20]. The Hxk2-wrf enzyme has been reported to lack gene regulatory activity [18], as judged by the derepression of invertase. We have been unable to reproduce that finding. Cells with the *hxk2-wrf* allele showed wild-type levels of invertase repression in our assay (Fig 1D). We have sequenced the *hxk2-wrf* and confirmed that it contains the deletion of residues 6–15 that are reported to cause a loss of regulatory function. Our results are consistent with those reported from the Botstein lab showing that deletion of amino acids 1–15 in Hxk2 had little effect on invertase expression [21]. This discrepancy may be attributable to the use of S288c and W303 yeast strains in different laboratories. Our results reported here and those reported earlier [22] indicate that *HXK2* mutations do not have a direct correlation between catalytic activity and catabolite repression. In contrast and consistent with our earlier study [8], we find that loss of catalytic activity of Hxk2 strongly correlates with 2DG resistance (Table 3).

**Table 3:** Activity of *HXK2* and variants.

HXK2 Allele	2DG Resistance % Growth in 0.1% 2DG	Catalytic Activity nmole/min/A.U.	Invertase Activity mU/OD
WT *HXK2*	20	4.317	53
*hxk2-WRF*	16	3.077	58
*hxk2-R423T*	61	0.127	45
*hxk2-D417G*	62	0.558	60
*hxk2-WCA*	73	0.452	134
*hxk2-G55V*	81	0.017	416
*hxk2Δ*	80	0.0	611
*hxk2-D211A*	81	0.0	1020

Previous studies indicated that deletion of *HXK2* led to an increase in the phosphorylation of the Snf1 kinase [8]. We analyzed the activation of Snf1 by western blotting using antibodies to total Snf1 and phosphorylated Snf1 (Fig 1E). Consistent with our earlier studies, deletion of *HXK2* led to an increase in phosphorylation of the Snf1 kinase especially in cells growing on glucose prior to addition of 2DG. All three of the strains with missense alleles in the *HXK2* gene showed an increase in Snf1 kinase phosphorylation.

### Recessive, missense alleles in PP1 phosphatase genes *REG1* and *GLC7* confer 2DG resistance

**Fig 2:**
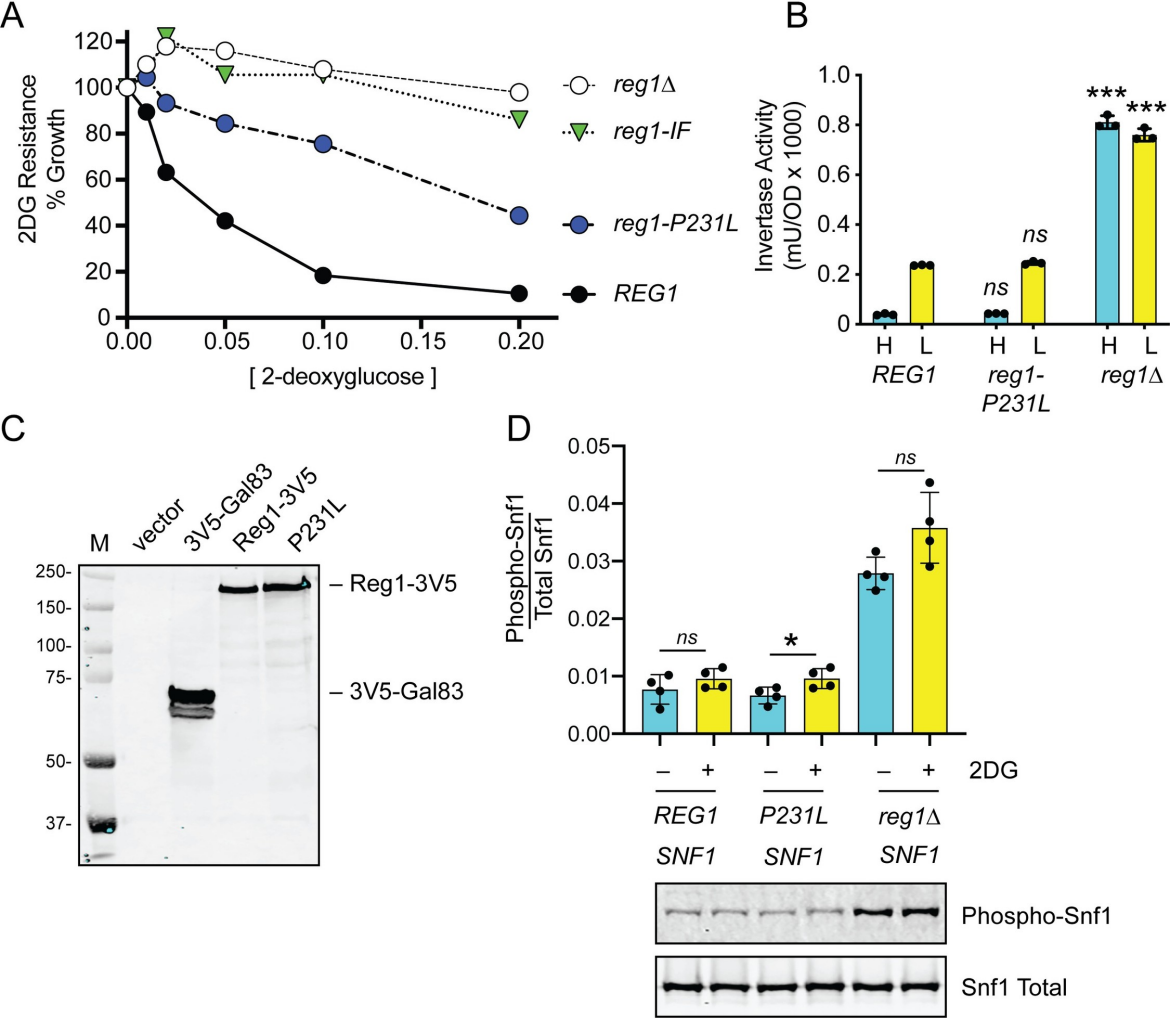
Amino acid substitution, Reg1-P231L, confers 2DG resistance. **A.** 2DG resistance assay of *reg1Δ* cells transformed with empty vector or plasmids expressing wildtype Reg1, Reg1-P231L or the Reg1-IF mutation known to disrupt interaction with Glc7 [28]. **B.** Invertase assays using cells from three independent cultures grown in 2% glucose (H) or two hours after shifting to 0.05% glucose (L) as indicated. The *REG1* genotype shown below. Individual data points are shown with the mean indicated with a solid bar. Values of the mutants were compared to wild type and statistical significance is shown. **C.** Western blot using V5 epitope-tagged proteins expressed from low-copy number plasmids. Positive (3V5-Gal83) and negative (vector) controls for the V5 antibody are shown. **D.** Western blot of HA-tagged Snf1 protein kinase using antibodies that detect total Snf1 protein or Snf1 protein phosphorylated on threonine 210. Quantitation of the western signals is shown in the bar graphs above the blot images. Samples were collected in quadruplicate from log cultures in the absence of 2DG or two hours after addition of 2DG to 0.1%.

Yeast PP1 phosphatase is composed of the catalytic subunit Glc7 associated with a number of alternative regulatory subunits [14]. The Glc7/Reg1 complex is the isoform of PP1 that participates in glucose repression by dephosphorylating the kinase Snf1 [24–26] and the transcription factor Mig1 [25, 27]. The *reg1-P231L* mutation found in our screen confers significant 2DG resistance in yeast when reconstituted on a low-copy CEN plasmid in a *reg1*Δ strain (Figs 2A and S2). For comparison, we also measured 2DG resistance in cells lacking the *REG1* gene (*reg1*Δ) and in cells expressing a variant of Reg1 (*reg1-IF*) that cannot bind to Glc7 [28, 29]. Cells lacking Reg1 or expressing Reg1-IF are almost completely resistant to 2DG. Cells lacking Reg1 are defective in catabolite repression [30] and express high levels of invertase even in the presence of glucose (Fig 2B). The Reg1*-*P231L protein did not show any defect in invertase repression and was present in cells at levels comparable to wild type Reg1 as judged by western blotting (Fig 2C). The Reg1 protein binds to the Snf1 kinase complex as judged by immunoprecipitation and two-hybrid analysis [29, 31]. The P231L substitution in Reg1 did cause some impairment in the interaction of Snf1 with the Reg1-P231L mutant by the yeast two-hybrid assay (S7 Fig). However, this substitution showed no significant effect on Snf1 activation loop phosphorylation in cells grown in glucose and two hours after addition of 2DG (Fig 2D). Addition of 2DG causes a small but consistent increase in Snf1 phosphorylation as we have reported previously [8]. Cells expressing Reg1-P231L showed a Snf1 phosphorylation level similar to wild type while deletion of *REG1* has a large effect on Snf1 activation consistent with earlier studies [24]. Loss of the *REG1* gene imposes a significant fitness cost [32] to cells growing on glucose (S8 Fig) resulting in pleiotropic phenotypes, slow growth and frequent occurrence of spontaneous suppressors [33]. In contrast, the *reg1-P231L* allele has a much lower fitness cost, does not cause a slow growth phenotype on glucose and does not derepress invertase, making it a potentially useful allele for further characterization of *REG1*.

**Fig 3:**
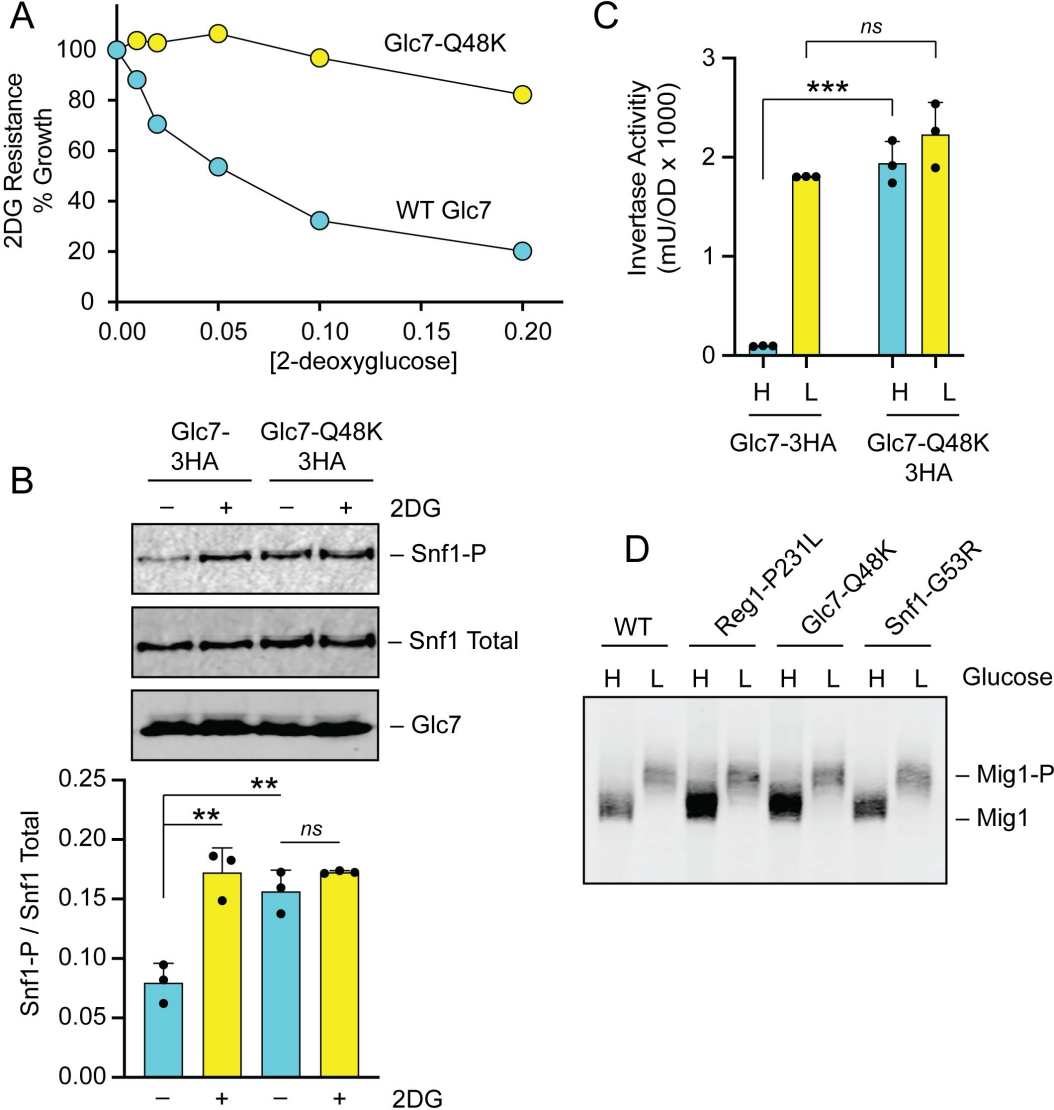
Amino acid substitution, Glc7-Q48K, confers 2DG resistance. **A.** 2DG resistance assay in *glc7Δ* cells containing plasmids to express HA-tagged wildtype Glc7 or HA-tagged Glc7-Q48K. **B.** Western blot of Snf1 kinase using antibodies to detect total Snf1 protein, Snf1 protein phosphorylated on threonine 210 (Snf1-P), or HA-tagged Glc7 protein. A plot of the ratio of phospho-Snf1 to total Snf1 is shown below for triplicate extracts grown prepared before and after incubation in 0.1% 2DG for 2 hours. A representative western blot is shown above. **C.** Invertase activity of three independent cultures grown in high glucose (H) or two hours after shifting to low glucose (L). Individual data points are shown with the mean indicated with a solid bar. Values statistically different from wild-type cells in the same glucose condition are indicated. **D.** Western blot of Mig1-3HA protein in cells grown in high (H) glucose or two hours after shifting to 0,05% glucose (L). Relevant genotypes are shown above. Migration of Mig1 and phosphorylated Mig1 are indicated.

The *GLC7* gene encodes the catalytic subunit of the PP1 phosphatase and is essential for viability [34]. To confirm that the *GLC7-Q48K* allele conferred 2DG resistance, we transformed a diploid heterozygous strain (*GLC7/glc7Δ*::*KAN*) with a low-copy plasmid encoding an HA-tagged *GLC7* with and without the Q48K mutation. After sporulation, we recovered viable haploid cells with both plasmid encoded *GLC7* genes and the chromosomal *glc7Δ*::*KAN* allele. We assayed both the wild type and the Q48K mutant strains and confirmed that the *glc7-Q48K* allele conferred 2DG resistance (Fig 3A and S2 Fig). This mutation did slightly weaken the yeast two-hybrid interaction of Glc7 and Reg1 (S7 Fig), and it resulted in an increase in basal phosphorylation of Snf1 (Fig 3B). The *glc7-Q48K* mutation also had a large effect on the regulation of invertase activity (Fig 3C). In cells expressing wild type Glc7-3HA, invertase expression was repressed in high glucose and induced when cells are shifted to low glucose (0.05%). In contrast, cells expressing Glc7-3HA with the Q48K substitution showed high levels of invertase expression in both high and low glucose. Repression of invertase expression in high glucose requires dephosphorylation of the Mig1 transcriptional repressor [27]. The phosphorylation state of Mig1 is readily discernable by western blotting, since phosphorylation of Mig1 causes significant reduction in gel mobility that was reversed by phosphatase treatment [35]. We tested whether any of the 2DG-resistant mutations in the PP1 phosphatase affected the phosphorylation state of Mig1. In wild-type cells, the phosphorylation of Mig1 was readily detected upon shifting cells to low glucose (Fig 3D). A similar pattern of Mig1 phosphorylation was observed for cells containing the *glc7-Q48K* and *reg1-P231L* alleles. Thus, the two missense mutations in the PP1 complex had no detectable effect on basal Mig1 phosphorylation in cells growing on high glucose, and yet these caused very different effects on catabolite repression of *SUC2*. These data suggest that some other step possibly downstream of Mig1 is differentially regulated in the strains bearing the *glc7-Q48K* and *reg1-P231L* alleles.

### Dominant allele of *SNF1* confers 2DG resistance

**Fig 4:**
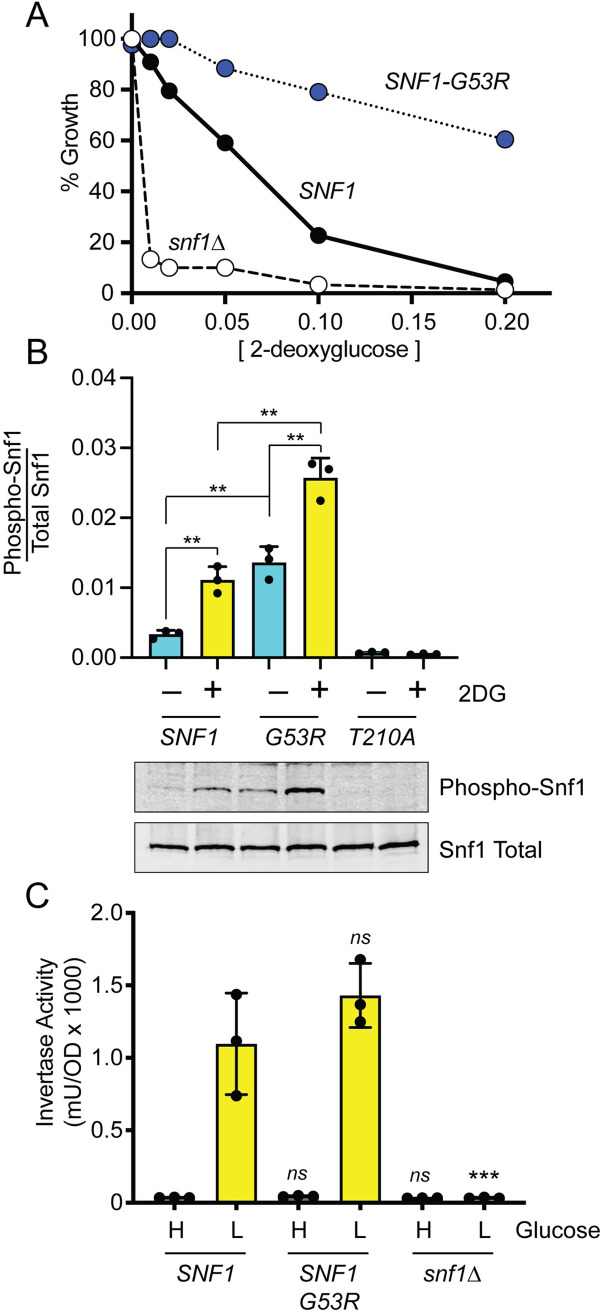
Amino acid substitution, Snf1-G53R, confers 2DG resistance. **A.** 2DG resistance assay in *snf1Δ* cells transformed with plasmids expressing either HA-tagged wild-type Snf1, HA-tagged Snf1-G53R, or no Snf1 (*snf1Δ*). **B.** Western blot of HA-tagged Snf1 protein kinase using antibodies that detect total Snf1 protein or Snf1 protein phosphorylated on threonine 210. Cells were harvested in mid-log phase or two hours after addition of 2DG to 0.1% as indicated. Quantitation of the western signals is shown in the bar graphs above the blot images. **C.** Invertase activity was measured in three independent cultures grown in high glucose (H) or two hours after shifting to 0.05% glucose (L). Values statistically different from wild-type values in the same glucose condition are indicated.

In our screen for 2DG-resistant mutations, we characterized 28 mutations. Twenty-seven were recessive mutations and one was dominant. When we sequenced the genome containing the one dominant allele, we identified the missense allele, *SNF1-G53R*, as the primary candidate responsible for 2DG resistance (Table 2). This allele of *SNF1* has been isolated previously as a dominant mutation that improves Snf1 kinase function in the absence of its gamma subunit, Snf4 [16]. To confirm that the *SNF1-G53R* mutation conferred 2DG resistance, *snf1Δ* cells were transformed with plasmids expressing wild type *SNF1* with a triple HA tag at the C-terminus [24], the *SNF1-G53R* allele, or empty vector. As we have previously shown, cells lacking a *SNF1* gene (*snf1Δ*) are hypersensitive to 2DG [8]. Cells with the *SNF1-G53R* were resistant to 2DG relative to wild-type cells (Fig 4A), consistent with the idea that *SNF1-G53R* is a hyperactive *SNF1* allele.

The phosphorylation status of the Snf1 kinase activation loop is a good proxy for determining Snf1 kinase activity [24]. We next examined whether the G53R substitution affected the phosphorylation status of the Snf1 activation loop using antibodies specific for Snf1 phosphorylated on threonine 210. The Snf1 kinase with the G53R substitution showed increased basal levels of threonine 210 phosphorylation in cultures grown in high glucose and a much greater activation in response to addition of 2DG (Fig 4B). As a control for antibody specificity, we show that the phospho-Snf1 antibody fails to detect any phosphorylated Snf1 in cells expressing the Snf1-T210A allele. When we analyzed the effect of the G53R substitution on glucose repression, we found that invertase expression was not significantly affected by this mutation (Fig 4C). A similar result with the *SNF1-G53R* allele was reported previously [16]. A known substrate of the Snf1 kinase is the transcriptional repressor Mig1. Cells expressing Snf1-G53R did not show altered phosphorylation of the Mig1 protein (Fig 3D). Thus, similar to what we observed for the *REG1* and *GLC7* mutations, the mechanism by which *SNF1-G53R* confers 2DG resistance does not seem to involve changes in Mig1-regulated gene expression.

### Mutations in Hxk2 and Snf1 pathway genes protect Hxt3 from endocytosis

**Fig 5:**
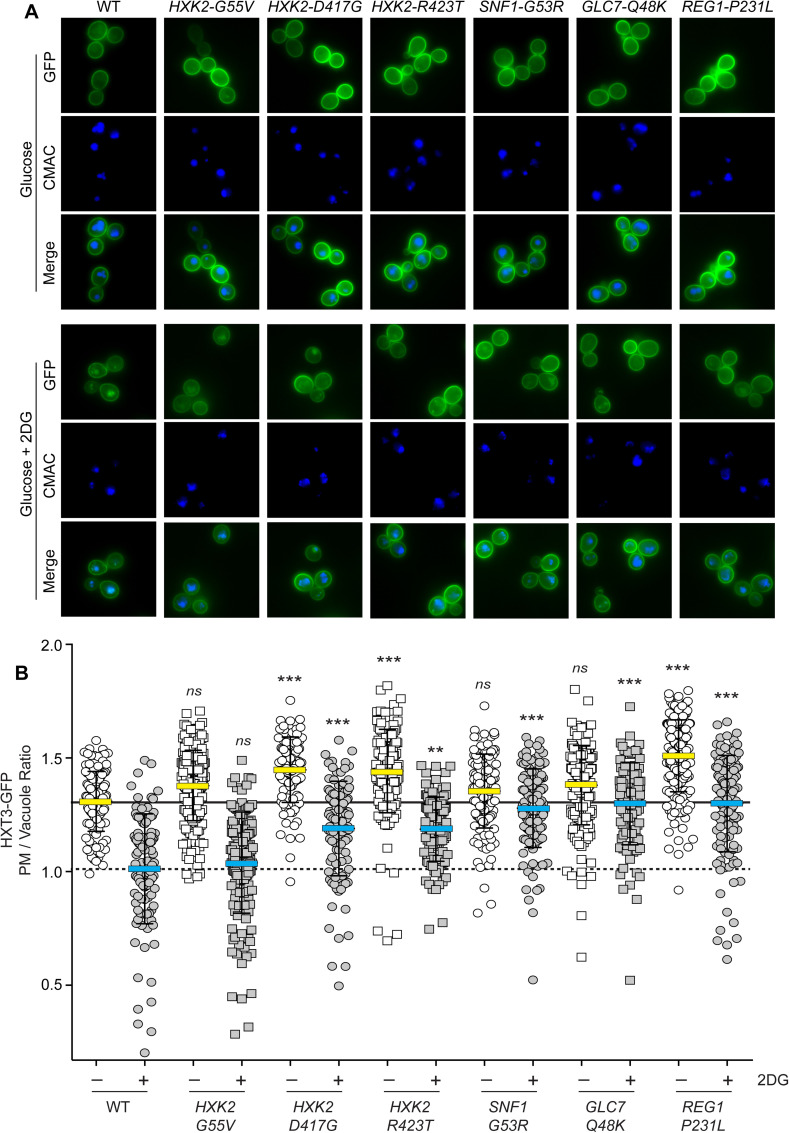
HXT3-GFP localization. **A.** Wild-type cells (WT) or cells with the indicated mutations with integrated Hxt3-GFP were stained with CMAC blue to identify the vacuole. Images were captured during growth on glucose or two hours after addition of 2DG. **B.** Quantitation of GFP fluorescence for at least 150 cells is plotted for each cell as the ratio of plasma membrane (PM) fluorescence to vacuolar fluorescence. The mean value for cells grown in glucose is marked with a yellow bar with the mean value for wild-type cells indicated as a solid reference line across the graph. The mean value for cells treated with 2DG is marked with a blue bar with the mean value for wild-type cells treated with 2DG is indicated with a hashed line as a reference value across the graph. Fluorescence values statistically different from wild type, where all untreated cells are compared to untreated WT and all 2DG-treated cells are compared to the 2DG-treated WT control.

In our earlier study of the mechanism of 2DG toxicity, we found that 2DG promoted the endocytosis of the high-capacity glucose transporters Hxt1 and Hxt3 [10]. The endocytosis of these glucose transporters was dependent on the arrestins Rod1 and Rog3. Phosphorylation of these α-arrestins by Snf1 impaired their ability to stimulate endocytosis of the Hxts in response to 2DG. The wild-type, 2DG-sensitive yeast strain (MSY1333; Table 4) that we used to select new spontaneous mutations contained an integrated copy of *HXT3-GFP* gene allowing us to track the localization of this glucose transporter by fluorescence microscopy. We measured the localization of Hxt3-GFP in our wild-type strain either grown in glucose or 2 hours after exposure to 2DG. Consistent with our earlier report, we found that 2DG promoted endocytosis of Hxt3-GFP (Fig 5A). This effect was quantified by measuring Hxt3-GFP fluorescence in individual cells and plotting the ratio of the fluorescence intensity at the plasma membrane relative to that of the vacuole (Fig 5B). When we analyzed Hxt3-GFP fluorescence in cells containing 2DG-resistant mutations, we found a significant increase in the plasma membrane localization of Hxt3-GFP for the cells expressing Hxk2-D417G, Hxk2-R423T, Snf1-G53R, Glc7-Q48K and Reg1-P231L. Curiously, the cells expressing Hxk2-G55V did not show any significant retention of Hxt3-GFP at the plasma membrane. Thus, the three Hxk2 mutations isolated in this study appear to have different effects in HXT3-GFP localization. The finding that all three mutations in the Snf1 pathway (*SNF1-G53R*, *glc7-Q48K and reg1-P231L)* promoted retention of Hxt3-GFP at the plasma membrane is consistent with our earlier finding that the Snf1 kinase plays an important role in regulating endocytosis of the Hxt3 protein. The mechanism by which some of the *HXK2* alleles regulated HXT3-GFP localization is not yet known.

**Table 4:** Yeast strains.

Strain	Genotype
MSY1333	*MATα ura3 leu2 his3 HXT3-GFP*::*His3MX*
MSY939	*MATα ura3-52 leu2Δ1 his3Δ200 trp1Δ63 reg1Δ*::*KAN snf1Δ10*
RG26132	*MAT****a****/α ura3Δ0/ura3Δ0 leu2Δ0/leu2Δ0 his3Δ1 his3Δ1 lys2Δ0/LYS2 met15Δ0/MET15 glc7Δ*::*KAN/GLC7*
MSY1255	*MAT****a*** *ura3Δ0 leu2Δ0 his3Δ1 met15Δ0 hxk2Δ*::*KAN*
MSY1477	*MAT****a*** *ura3Δ0 leu2Δ0 his3Δ1 met15Δ0 hxk1Δ*::*KAN hxk2*::*KAN glk1Δ*::*KAN*
MSY1155	*MAT****a*** *ura3-52 leu2 trp1-901 his3-Δ200 gal4Δ gal80Δ LYS2*::*GAL1-HIS3 met2*::*GAL7-lacZ*
MSY930	*MAT****a*** *ura3Δ0 leu2Δ0 his3Δ1 met15Δ0 reg1Δ*::*KAN*
MSY1508	*MATα ura3 leu2 his3 HXT3-GFP*::*His3MX hxk2-D417G*
MSY1509	*MATα ura3 leu2 his3 HXT3-GFP*::*His3MX hxk2-G55V*
MSY1510	*MATα ura3 leu2 his3 HXT3-GFP*::*His3MX hxk2-R423T*
MSY1511	*MATα ura3 leu2 his3 HXT3-GFP*::*His3MX reg1-P231L*
MSY1512	*MATα ura3 leu2 his3 HXT3-GFP*::*His3MX glc7-Q48K*
MSY1513	*MATα ura3 leu2 his3 HXT3-GFP*::*His3MX SNF1-G53R*
MSY1535	*MAT****a*** *ura3-52 leu2Δ1 his3Δ200 dog1Δ-dog2Δ*::*HIS3*
MSY1537	*MAT****a*** *ura3Δ0 leu2Δ0 his3Δ1 met15Δ0 dog1Δ-dog2Δ*::*HIS3 reg1Δ*::*KAN*
MSY1538	*MATα ura3Δ0 leu2Δ0 his3Δ1 lys2Δ0 dog1Δ-dog2Δ*::*HIS3 hxk2Δ*::*KAN*
MSY1212	*MAT****a*** *ura3-52 leu2Δ1 his3Δ200*
MSY1282	*MAT****a*** *ura3 leu2 his3 met15Δ0 rog3Δ*::*KAN rod1Δ*::*KAN*
MSY1542	*MAT****a*** *ura3 leu2 his3 rog3Δ*::*KAN rod1Δ*::*KAN HXT1-GFP*::*LEU2 dog1/2Δ*::*HIS3*
MSY1543	*MATα ura3 leu2 his3 met15Δ0 lys2Δ0 rod1Δ*::*KAN rog3Δ*::*KAN hxk2Δ*::*KAN dog1/2Δ*::*HIS3*
MSY1544	*MATα ura3 leu2 his3 rod1Δ*::*KAN rog3Δ*::*KAN hxk2Δ*::*KAN*
MSY1538	*MATα ura3Δ0 leu2Δ0 his3Δ1 lys2Δ0 hxk2*::*KAN dog1/2Δ*::*HIS3*
MSY1470	*MATα ura3Δ0 leu2Δ0 his3Δ1 met15Δ0 glk1Δ*::*KAN hxk2Δ*::*KAN*
MSY1471	*MATα ura3Δ0 leu2Δ0 his3Δ1 glk1Δ*::*KAN*
MSY1472	*MAT****a*** *ura3Δ0 leu2Δ0 his3Δ1 hxk1Δ*::*KAN hxk2Δ*::*KAN*
RG5867	*MAT****a*** *ura3Δ0 leu2Δ0 his3Δ1 met15Δ0 hxk1Δ*::*KAN*
MSY1478	*MATα ura3Δ0 leu2Δ0 his3Δ1 met15Δ0 hxk1Δ*::*KAN glk1Δ*::*KAN*

### Transcriptional response to acute carbon source stress

**Fig 6:**
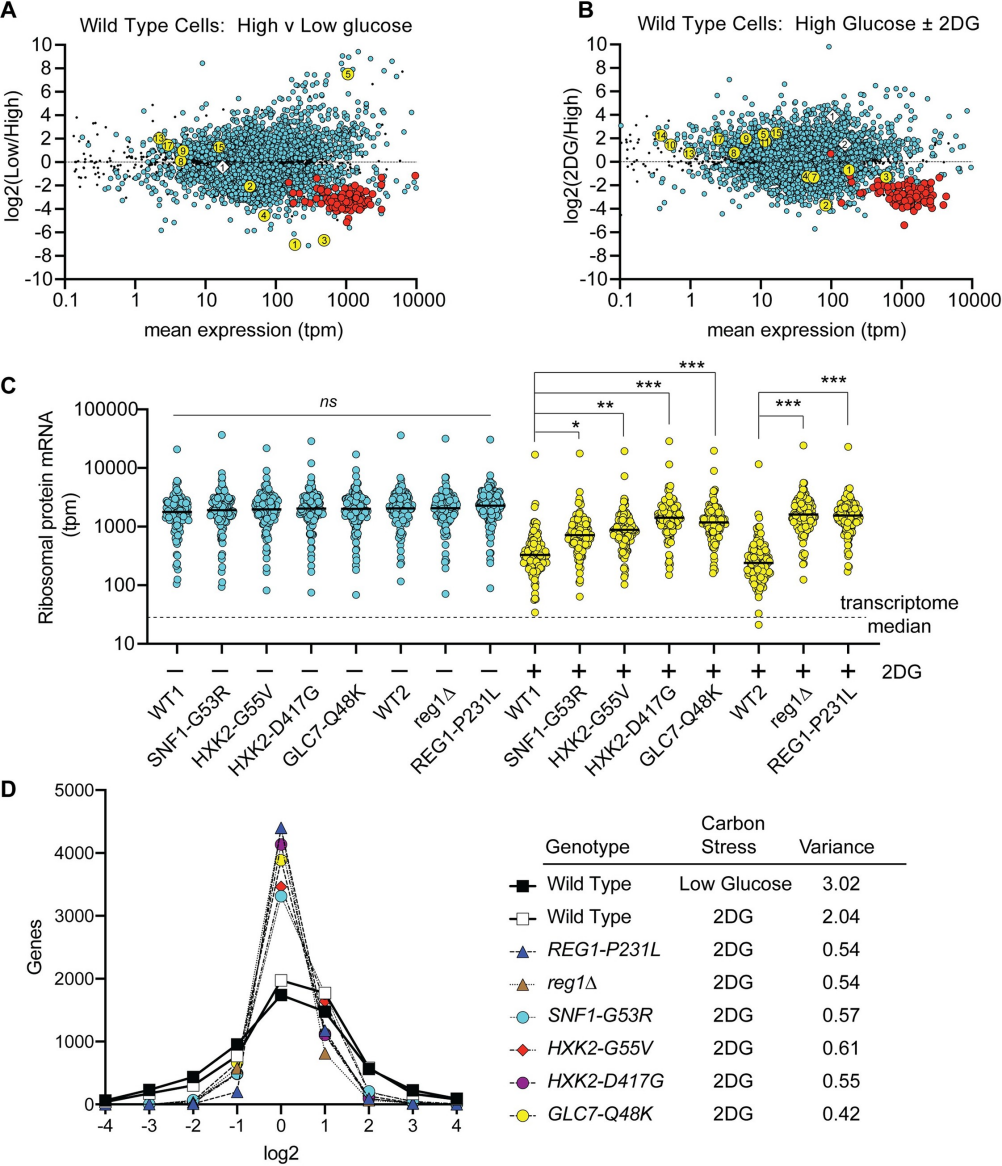
Transcriptomic response of wild-type and 2DG resistant mutants undergoing carbon source stress. RNA was extracted from four independent yeast cultures of wild-type cells (MSY1212) grown to mid-log phase in synthetic complete media with 2% glucose and two hours after shifting to 0.05% glucose **(A)** or two hours after addition of 2DG to 0.1%. **(B)**. RNA abundance was quantified and is displayed here for 5917 mRNAs as the log2 ratio of the mean expression values (tpm) on the y-axis and the mean expression values under both conditions on the x-axis. Expression levels that show a statistically significant change using an adjusted p-value with a false-discovery rate threshold of 0.01 or lower are shown as colored circles. Those not meeting this threshold are shown as smaller black dots. Ribosomal protein genes (red circles), hexose transporter genes (yellow circles) and *DOG1* and *DOG2* genes (white diamonds) that meet the false discovery rate threshold are indicated. **(C)** RNA was extracted from three independent yeast cultures with the indicated genotypes after growth to mid-log phase in high glucose or two hours after addition of 2DG to 0.1%. Mean expression levels of the ribosomal protein gene mRNAs are plotted with a student t-test used to determine statistically significant changes in expression levels (*, p<0.05; **, p<0.01; ***, p<0.001). **(D)** The scale of the transcriptomic response to carbon source stress in wild type and 2DG-resistant mutants is plotted as log2 ratio of mean values (bin size of 1.0) versus gene number. Variance of all log2 values is shown for each strain and condition plotted.

In order to better understand the global transcriptional response to carbon source stress, we measured the abundance of mRNAs in cells using RNAseq. Total RNA was harvested from wild-type cells growing in synthetic complete media with 2% glucose as the carbon source or from cells shifted for two hours to either low glucose (0.05% glucose) or 2% glucose plus 0.1% 2DG. RNA samples were prepared from at least three to four independent cultures and at least fifty million reads were mapped for each sample. Abundance of each mRNA was determined using kallisto software [36] and is expressed as transcripts per million mapped reads (tpm). We plotted the mean expression of the mRNA under both conditions on the x-axis and on the y-axis, the log2 ratio of the mean tpm values for the two conditions (Fig 6A and 6B). This allows for simultaneous visualization of the change in expression in response to these carbon source stresses as well as the relative abundance of each mRNA. One striking transcriptional response in cells undergoing both glucose starvation and 2DG stresses was the ~10-fold reduction in the abundance of ribosomal protein mRNAs compared to cells growing in 2% glucose (red circles). Since ribosome assembly is a key rate limiting step of cellular proliferation, this down-regulation of ribosomal protein mRNA may reflect a reduced growth rate in the presence of unfavorable conditions. While the reduction in ribosomal protein mRNA abundance was consistently observed in wild type, 2DG-sensitive cells, this response was significantly lessened in all of 2DG-resistant strains examined (Fig 6C). Thus, cells that have developed spontaneous resistance to 2DG somehow reprogram their transcriptional response to avoid the severe downregulation of ribosomal protein mRNAs that occurs when cells are challenged with 2DG-treatment. This observation is an exciting and previously unknown regulatory feature in response to 2DG that likely has a significant role to play in developing 2DG-resistance. When cells undergo carbon source stress, either low glucose or addition of 2DG, one third of the yeast transcriptome (~2000 genes) exhibits at least a 2-fold change in expression. In contrast, the 2DG-resistant strains exhibit a much smaller transcriptional response to 2DG with the great majority of the genes (~5000) exhibiting less than a two-fold change in gene expression (S9 Fig). The great reduction in transcriptomic response is evident in the greatly reduced variance of the log2 ratios of all mRNAs over the entire transcriptome in all of the 2DG resistant strains isolated in this study (Fig 6D).

Next, we specifically examined the *HXT* genes, since their abundance and localization has been linked to 2DG resistance [10]. When cells were shifted to low glucose, the mRNA abundance for the high capacity transporters (*HXT1*, *HXT2*, *HXT3 and HXT4*) decreased dramatically, while the high affinity transporters (*HXT5* and *HXT6*) showed increased mRNA abundance (Fig 6A; yellow circles). This response to reduced glucose abundance was consistent with what has been previously for these *HXT* genes using lacZ-reporter assays [37, 38]. In contrast, when cells are exposed to 2DG, they responded by decreasing the abundance of the highly expressed *HXT* genes but do not induce high level expression of any of the high affinity *HXT* genes. Therefore, cells exposed to 2DG respond both by decreasing the expression of *HXT* mRNA (Fig 6B) and by removing *HXT* proteins from the plasma membrane (Fig 5).

### Contribution of the DOG phosphatases to the 2DG-resistant phenotypes

**Fig 7:**
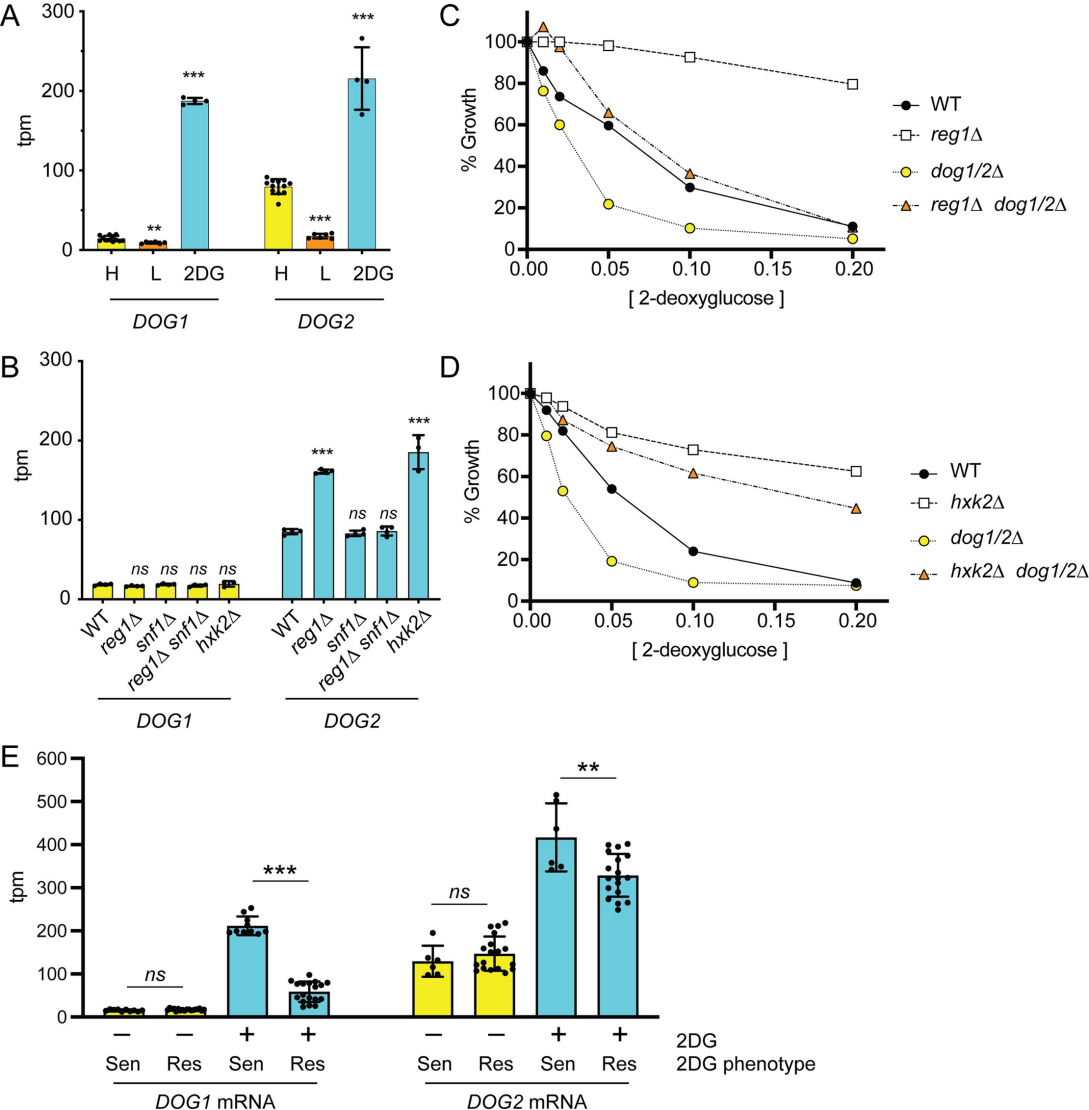
2DG-resistance does not require the DOG phosphatases. **A-B.** Messenger mRNA abundance for the *DOG1* and *DOG2* genes was measured by RNAseq in mulitple independent replicates and plotted as transcripts per million mapped reads (tpm). Values statistically different from wild type in high glucose are shown. **A.** RNA was isolated from cells grown in high glucose (H), two hours after shifting to 0.05% glucose (L), or two hours after addition of 2DG to a final concentration of 0.1%. **B.** Abundance of *DOG1* and *DOG2* mRNA is shown for wild-type cells, *reg1Δ*, *snf1Δ*, *reg1Δ snf1Δ* and *hxk2Δ* cells grown in high glucose. **C and D.** 2DG resistance assays for wild-type (WT) cells and cells with the indicated genotypes. **E.** Transcriptional response to 2DG was measured by RNAseq in wild type cells sensitive to 2DG (Sen) and 2DG-resistant (Res) strains (*SNF1-G53R*, *HXK2-G55V*, *HXK2-D417G*, *GLC7-Q48K*, *REG1-P231L and reg1Δ*). RNA was harvested in triplicate from cells grown to mid-log in 2% glucose (–) or two hours after addition of 2DG (+) as indicated. The expression level of the *DOG1* and *DOG2* mRNAs (tpm) is plotted with statistically significant differences indicated.

Overexpression of the DeOxyGlucose (DOG) phosphatases 1 and 2 confers resistance to 2DG [13, 15]. We sought to determine what role these phosphatases played in the 2DG resistance caused by the mutations identified in this study. First, we examined the mRNA levels of the *DOG1* and *DOG2* genes using RNAseq. We found that under basal conditions of growth to mid-log phase in synthetic complete media with high glucose, *DOG2* mRNA is expressed at higher levels than *DOG1* mRNA (Fig 7A). Upon shifting cells to low-glucose media for two hours, conditions that activate the Snf1 kinase [24], we found a significant reduction in mRNA abundance for both *DOG* mRNAs. More germane to this study, both *DOG* gene mRNAs showed marked increases following a two-hour exposure to 2DG. We also examined the abundance of the *DOG* mRNAs in wild type compared to *reg1Δ* and *hxk2Δ* cells. Both deletions confer 2DG resistance, and both lead to a significant increase in *DOG2* but not *DOG1* mRNA abundance (Fig 7B). Increased expression of the *DOG2* mRNA observed in the *reg1Δ* cells was dependent on the Snf1 kinase, since the increase was not observed in the double *reg1Δ snf1Δ* cells. These findings suggested that the underlying mechanism of 2DG resistance was due to increased expression of the Dog2 phosphatase. We tested this idea by measuring 2DG resistance in strains that lacked the *REG1* or *HXK2* genes with or without a deletion covering both the *DOG1* and *DOG2* genes. These genes are adjacent on chromosome 8 and we replaced both genes with the *HIS3* gene to create the double *DOG* deletion (*dog1/2Δ*). As shown previously [8], deletion of *REG1* confers robust resistance to 2DG (Fig 7C). Deletion of both *DOG1* and *DOG2* genes makes cells more sensitive to 2DG, a result recently reported by the Leon lab [15]. When *REG1* was deleted from the *dog1/2Δ* strain, the cells became more resistant to 2DG. Thus, deletion of *REG1* was able to confer 2DG resistance independently of the *DOG1* and *DOG2* genes. An alternative view of this experiment is to note that deletion of the *DOG1* and *DOG2* genes reduced the 2DG resistance of the *reg1Δ* strain. Therefore, the DOG phosphatases must contribute to some of the 2DG resistance observed in the *reg1Δ* strain. A similar analysis was performed with cells lacking the *HXK2* gene (Fig 7D). Deletion of the *HXK2* gene conferred robust 2DG resistance and further deletion of the *DOG1* and *DOG2* genes only slightly reduced that resistance. Therefore, the DOG phosphatases play a much smaller role in the resistance conferred by deletion of *HXK2*. These data suggest that deletion of the *REG1* and *HXK2* genes may confer 2DG resistance by distinct mechanisms and while the DOG phosphatases contribute to some degree to these phenotypes, there must be DOG-independent mechanisms of 2DG resistance. Finally, we examined the contribution of DOG gene expression in the 2DG-resistant strains isolated in this study. We harvested RNA from wild type, 2DG sensitive cells and from 6 2DG-resistant strains before and after addition of 2DG (Fig 7E). While the 2DG-resistant strains were able to induce *DOG2* expression in response to 2DG addition, the transcriptional induction was significantly reduced. This muted transcriptional response to 2DG was more severe at the *DOG1* locus. The reduced transcriptional response to 2DG at the *DOG1* and *DOG2* loci in the resistant strains is consistent with the muted transcriptional response in the resistant strains observed of the entire transciptome (Figs 6D and S9). Taken together, the data reported here indicate that increased expression of the DOG phosphatases is not critical to acquisition of 2DG resistance.

### Genetic requirements for 2DG resistance conferred by the *SNF1-G53R* allele

**Fig 8:**
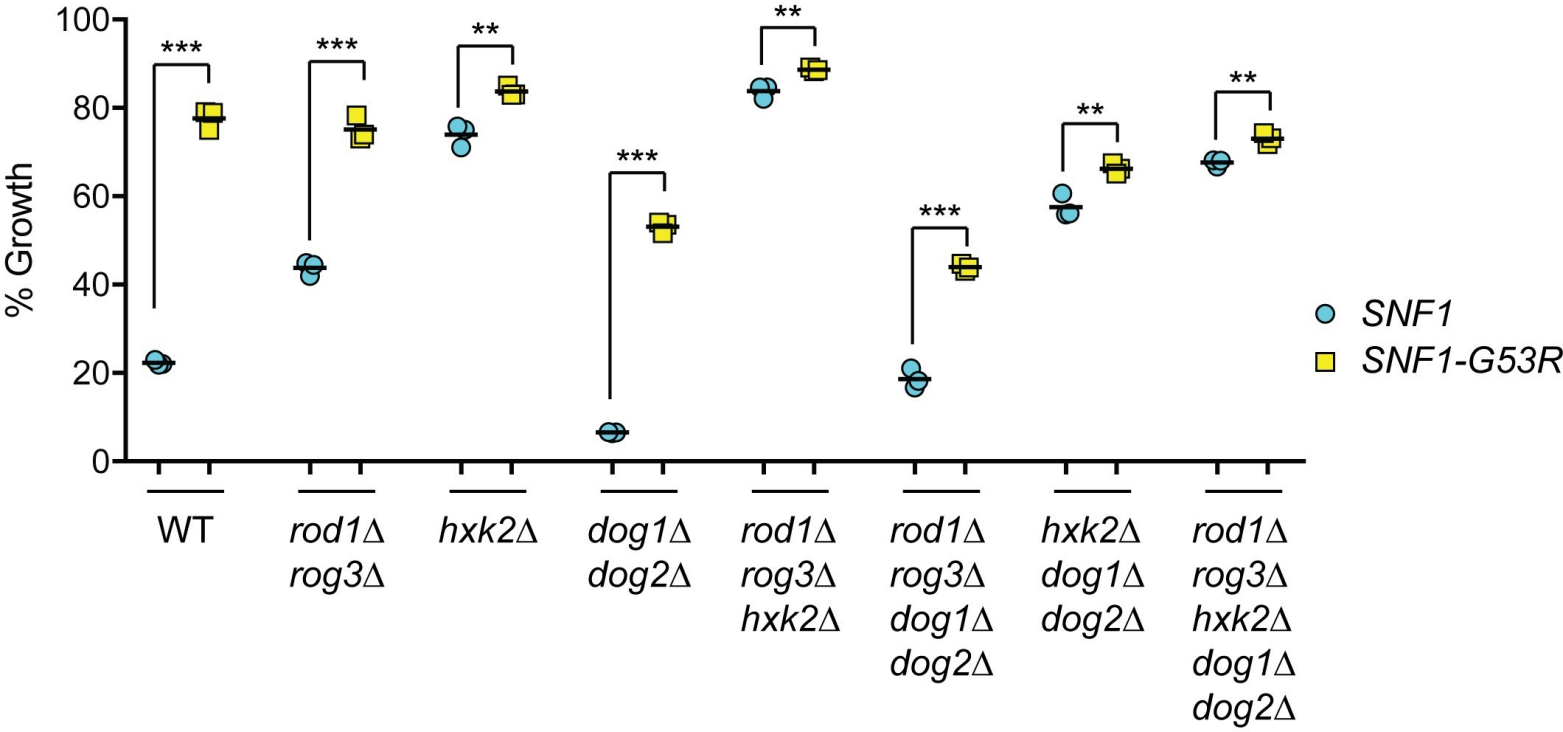
Genetic Requirements for Snf1-G53R-mediated 2DG Resistance. Cells with the indicated genotypes were transformed with low-copy plasmids expressing either wild type Snf1 (blue circles) or Snf1-G53R (yellow squares). Cells were grown in triplicate overnight in SC media with 2% glucose in the absence or presence of 0.1% 2DG. Growth is expressed as a percentage relative to growth in the absence of 2DG.

In our screen for 2DG-resistant mutants, we uncovered a single dominant allele, *SNF1-G53R*. We took advantage of the dominant inheritance of the 2DG resistance phenotype to further test the genetic requirements for 2DG resistance. Our data suggest multiple mechanisms for 2DG resistance act in parallel to confer resistance. Regulation of arrestin-mediated endocytosis of the hexose transporters plays an important role, since deletion of *ROD1* and *ROG3* confers resistance to 2DG [10]. Yet the Snf1-G53R kinase conferred additional resistance in the *rod1Δ rog3Δ* cells (Fig 8), proving that Rod1- and Rog3-independent mechanisms are also operative. In a similar vein, the DOG phosphatases play an important role in 2DG resistance, since deletion of those genes makes cells more sensitive to 2DG (Figs 7C, 7D and 8). Nonetheless, the Snf1-G53R kinase conferred 2DG resistance in the double *dog1/2Δ* delete strain, proving that Snf1 can mediate 2DG resistance via a mechanism that is independent of the DOG phosphatases. We next made strains with deletions in multiple pathways (Rod1 and Rog3 arrestins, Dog phosphatases and Hxk2) to see if we could generate a strain in which the Snf1-G53R kinase could no longer confer resistance. In all combinations of deletions, the Snf1-G53R kinase was able to confer a small but statistically significant level of resistance (Fig 8). Thus, it seems likely that at least one additional Snf1-regulated pathway for 2DG resistance remains to be identified.

## Discussion

The use of 2DG and other metabolic inhibitors as potential cancer therapeutics makes it important to have a deep understanding of their mechanisms of action and the means by which cells acquire resistance. This latter concern is particularly critical for consideration in combinatorial clinical approaches that will block multiple metabolic pathways to more potently inhibit cancer cell progression. In this study, we avoided the use of alternative carbon sources and selected for 2DG resistance in cells growing on glucose as the carbon source, since this would be a growth condition that more closely mimics the plasma glucose used by human tumors. We isolated numerous resistant strains that all had mutations with the potential to alter the activity and or target selection of the Snf1 kinase signaling pathway. We identified multiple missense alleles in the *HXK2* gene that confer 2DG resistance and greatly diminish catalytic activity. We have previously shown that deletion of *HXK2* leads to increased phosphorylation of the Snf1 kinase [8] and the missense alleles identified here also lead to increased phosphorylation of Snf1 (Fig 1E). We identified two novel amino acid substitutions in the Glc7 and Reg1 proteins that constitute the isoform of the yeast PP1 phosphatase known to inactivate Snf1 kinase [24, 30, 39]. Cells expressing Glc7-Q48K showed increased activation of Snf1 (Fig 3B) and increased invertase activity (Fig 3C) while cells expressing the Reg1-P231L protein did not show either of these effects (Fig 2B and 2D). It is possible that cells expressing the Reg1-P231L protein do not affect PP1 dephosphorylation of the Snf1 kinase but affect the ability of PP1 to target some other substrate. Further characterization of the *REG1-P231L* allele may allow better understanding of Reg1 protein function in 2DG resistance. We also isolated a dominant, activating allele of *SNF1* itself. The amino acid substitution G53R in the Snf1 kinase protein was originally identified in a screen for hydroxylamine-mediated mutations in the *SNF1* gene that conferred Snf1 kinase activity in the absence of the kinase gamma subunit [16]. A similar screen using PCR-mediated mutagenesis of the Snf1 gene identified *SNF1-L183I*. Both of these substitutions, G53R and L183I, activate Snf1 in the absence of the gamma subunit, both confer resistance to 2DG, and yet neither increases invertase activity (Fig 4A and [40]). We have previously shown that different stresses, such as glucose starvation and alkaline pH stress, can each activate Snf1 yet result in different signal transduction outputs [41]. While the α -arrestins Rod1 and Rog3 are known downstream targets of Snf1 following 2DG treatment [10], they cannot be the only Snf1 important substrates in response to 2DG since we detect significant 2DG resistance induced by Snf1-G53R in the absence of the Rod1 and Rog3 proteins (Fig 8). Thus the full scope of targets of the Snf1 signaling pathway in response to 2DG must be broader and will be an exciting future direction for the field.

We analyzed the six 2DG-resistant strains isolated in this study and looked for a common feature that could constitute a shared adaptive response to 2DG. Wild type cells respond to the addition of 2DG by withdrawing the high capacity glucose transporters Hxt1 and Hxt3 from the plasma membrane and directing them to the vacuole (Fig 5 and [10]). In five of the six 2DG-resistant strains, this redirection of Hxt3 to the vacuole is significantly reduced leaving more Hxt3 at the cell surface and allowing for added glucose uptake in the presence of 2DG. We show here for the first time that addition of 2DG leads to a robust reprogramming of transcription with over 2000 genes changing expression by at least two-fold (Fig 6B and 6D). This transcriptome wide response to 2DG is significantly reduced in all six of the 2DG resistant strains isolated in this study and is clearly apparent in the ribosomal protein mRNAs (Fig 6C), in the DOG mRNAs (Fig 7E) and transcriptome wide (Fig 6D). Taken together, these data suggest that the growth inhibition by 2DG observed wild type cells might be caused by acellular ‘over-reaction’ to the accumulation of 2DG-6P, triggering endocytosis of the glucose transporters and rewiring cellular transcriptional networks, that ultimately leads to reduced fermentation of glucose and cellular starvation. The adaptive response shared by all of the 2DG-resistant strains may simply be to not over-react. This idea is supported by our findings that the withdrawal of Hxt3 from the plasma membrane and the transcriptional response are both significantly reduced in the 2DG-resistant strains. Strains that maintain transporters for glucose uptake and a transcriptional profile better suited to the high glucose environment have improved growth despite the internal accumulation of 2DG-6P.

Yeast express three hexokinase enzymes named Hxk1, Hxk2 and Glk1. All three can phosphorylate glucose, and deletion of all three is needed to significantly block glucose fermentation (S3A Fig). We show that both Hxk1 and Hxk2 are able to phosphorylate 2DG to produce 2DG-6P (S4 and S5 Figs and Table 2) yet only *HXK2* mutations confer resistance to 2DG. Several metabolic enzymes in yeast have been shown to possess additional “moonlighting” activities including transport to the nucleus and regulation of gene expression [42]. Hxk2 is just such an enzyme with a surprisingly large number of other activities that have been attributed to it, including short and long term glucose repression [20], nuclear localization [43], autophosphorylation [44], protein kinase activity [45], promoter binding and gene repression [46], regulation of Ras localization and apoptosis [47], and lifespan regulation [48]. Which if any of these other Hxk2 activities participates in 2DG resistance is not known. Humans express four distinct hexokinase enzymes that differ in tissue specific expression and in their sensitivity to product inhibition [49]. Tissues such as liver and pancreas that need to sense changes in plasma glucose concentrations express hexokinase IV, an isoform that is resistant to inhibition by accumulation of glucose-6-phosphate. Whether the Hxk1 and Hxk2 enzymes in yeast have differences in their inhibition by glucose-6-phosphate or 2DG-6P is not known but might explain their different abilities to confer resistance to 2DG.

This study demonstrates that multiple independent and parallel pathways are operating in the resistance to 2DG. Up-regulation of the DOG phosphatases and enhanced plasma membrane retention of the hexose transporters are important determinants of 2DG resistance. Even so, there must be other mechanisms that remain to be discovered. When endocytosis of Hxt3 is diminished by deletion of the *ROD1* and *ROG3* genes [10], we found that Snf1-G53R still promoted additional 2DG resistance (Fig 8). Likewise, when both of the *DOG* phosphatase genes were deleted, Snf1-G53R promoted additional 2DG resistance. Even in strain lacking both arrestins and both DOG phosphatase genes, Snf1-G53R further increased in 2DG resistance. These data suggest that multiple pathways are operating in parallel and that at least one additional Snf1-dependent pathway remains to be discovered.

## Material and methods

### Yeast strains and growth conditions

The yeast strains used in this study were all derivatives of S288C. Yeast strains with specific gene deletions were generated in our laboratory or by the *Saccharomyces* Genome Deletion Project [50] and purchased from Thermo Fisher Scientific (Table 4). Cells were grown at 30°C using standard synthetic complete media lacking nutrients needed for plasmid selection [51]. All cells were grown with 2% glucose (g/100 ml) unless stated otherwise.

### Selection of spontaneous 2DG-resistant mutants

Spontaneous mutations that conferred 2DG resistance were selected in the haploid strain MSY1333 (Table 1). Approximately 2x10^7^ cells were spread on agar plates containing synthetic complete media with 2% glucose (g/100ml) and 0.1% 2-deoxyglucose. Plates were incubated at 30°C for 4–6 days, and colonies were isolated for further study.

### Whole Genome Sequencing analysis of 2DG-resistant mutants

Spontaneously 2DG-resistant strains were analyzed for mutations by whole genomic sequencing using pooled linkage analysis [52] when possible. Genomic DNA was extracted using a glass bead phenol extraction method [53]. Sequencing libraries were prepared using a modified Illumina Nextera protocol and multiplexed onto a single run on an Illumina NextSeq500 to produce 151-bp paired-end reads [17]. Sequencing produced an average depth of 10–20 million reads per sample. Reads were mapped against the reference genome of strain S288C and variants detected using CLC Genomics Workbench (Qiagen).

### Mutagenesis and Epitope Tagging of plasmid constructs

Oligonucleotide-directed mutagenesis was performed with Pfu polymerase, followed by digestion of the starting plasmid template with the restriction enzyme *DpnI* [54]. All mutations reported in the study were confirmed by DNA sequencing. The wild-type *REG1* and *HXK2* alleles as well as mutant alleles were modified to contain three copies of V5 epitope [55]. The Snf1 and Glc7 proteins were epitope tagged with three tandem copies of the HA epitope placed at the C-terminus of the open reading frame [24, 29]. All of these constructs were introduced to yeast cells on low-copy CEN plasmids [56] and were expressed from their respective native promoters. To create a yeast strain expressing HA-tagged Glc7-Q48K, a diploid yeast strain heterozygous at the *GLC7* locus (*GLC7/glc7Δ*::*KAN)* was transformed with *GLC7* plasmids under *LEU2* selection. After sporulation, haploid segregants that were KAN+ and LEU+ were collected. These haploid cells contained the *glc7Δ*::*KAN* allele on chromosome five but were viable due to the presence of HA-tagged *GLC7* or *glc7-Q48K* on a plasmid.

### 2-deoxyglucose resistance assays

2DG resistance was measured in liquid cultures as previously described [8]. Briefly, overnight cultures were diluted to A_600_ of 0.1 and grown in the absence of 2DG or in the presence of increasing concentrations of 2DG (0.01%, 0.02%, 0.05%, 0.1%, 0.2%). Cells were grown for 18hr at 30°C. Each A_600_ was measured, and cell growth was normalized to growth in the absence of 2DG for each strain.

### Enzyme assays

The invertase activity of log-phase cells grown in high glucose or 2 hours after shifting to low glucose media was quantitatively assayed used a colorimetric assay coupled to glucose oxidase [57]. Three independent cultures were assayed, and the mean value is plotted with standard error indicated. The units of invertase activity used were mU/OD, where 1 U equals one μmole glucose released per minute. Hexokinase enzyme activity was measured by coupling the phosphorylation of glucose to its oxidation by glucose-6-phosphate dehydrogenase [21, 22]. Assays were conducted in 100 μl reactions containing 50 mM Tris-Cl pH 7.4, 10 mM MgCl_2_, 5% glycerol (vol/vol) 1 mM ATP, 10 mM glucose, 0.3 U glucose-6-phosphate dehydrogenase (Sigma G7877) and 0.1 mM NADP^+^. Production of NADPH was determined in a 96-well plate reader by measuring absorbance at 340 nm using the extinction coefficient of 6,220 M^-1^ cm^-1^. Units of hexokinase activity were expressed as nmoles/min. Hexokinase enzymes were assayed in yeast whole cell extracts prepared from cells lacking all three yeast hexokinase genes (MSY1477) and expressing a single hexokinase from a low-copy plasmid.

### Protein extractions

Trichloroacetic acid (TCA) was utilized for extraction of the Reg1 using a modification of a method previously reported [58]. Log-phase cells (2.5 OD’s) were harvested and washed with cold water before being resuspended in 1 ml of cold sterile water. Next, 150 ul of 2N NaOH and 78.3 ul of 1.12 M β-mercaptoethanol were added, and samples incubated on ice for 15 minutes. To precipitate proteins, 150 ul of cold TCA (50%) was subsequently added and incubated for 20 more minutes on ice. Samples were centrifuged at 4°C for 5 minutes, and pellets were resuspended in 50 ul TCA sample buffer (80 mM Tris-Cl pH 8.0, 8 mM EDTA, 120 mM DTT, 3.5% SDS, 0.29% glycerol, 0.08% Tris base, 0.01% bromophenol blue). Samples were incubated at 37°C for 30 minutes, and cell debris was removed by centrifugation prior to gel electrophoresis.

Glass bead extracts were prepared from liquid cultures grown to mid-log (OD_600_ of 0.6 to 0.8). 25 mL of cells were collected by centrifugation at 4°C at 3500 rpm and washed once in extraction buffer (20 mM Hepes pH 7, 0.5 mM EDTA, 0.5 mM DTT, 5 mM MgAc, 0.1 M NaCl, 10% glycerol). The cell pellet was resuspended in extraction buffer (4 packed cell volumes) supplemented with added protease inhibitors. Glass beads equal to the volume of the pellet were added, and the cells were shaken in an MP FastPrep-24 for three cycles of 20 seconds with 5 minutes of rest on ice between each cycle. Cell debris was removed by centrifugation at maximum speed for 5 minutes. Extracts were frozen and stored at -80°C.

### Western blotting

Western blotting techniques were modified from previous methods [29, 59]. Proteins tagged with the HA epitope (Snf1, Glc7) were detected with Anti-HA probe (Santa Cruz) diluted 1:2,000. Goat anti-mouse IRDye 800CW (Li-Cor) diluted 1:5,000 was used as the secondary antibody. For detection of phosphorylated Snf1, Phospho-AMPKalpha (Thr172) antibody (Cell Signaling) diluted 1:1,000 was used. Goat anti-rabbit IRDye 800CW (Li-Cor) was used as the secondary antibody at a 1:10,000 dilution. Proteins tagged with the V5 epitope (Hxk2, Reg1) were detected with the Anti-V5 probe (Invitrogen) diluted 1:1,000. Goat anti-mouse IRDye 680 (Thermo) diluted 1:10,000 was used as the secondary antibody. Blots were visualized using an Odyssey Infrared Imager (Li-Cor), and band quantification was performed using Odyssey software.

### Two-hybrid analysis

Two-hybrid interactions were analyzed as previously described [39]. Interactions between proteins were assessed by growth on medium containing 2% glucose lacking histidine, using strains with the *GAL1-HIS3* reporter integrated at the *LYS2* locus. The positive control plasmids used in this study encode the herpes simplex virus capsid protein 22a [60]. The entire open reading frame for the proteins Snf1 and Glc7 and their derivatives Snf1-G53R and Glc7-Q48K were expressed as a fusion to the Gal4 DNA binding domain in pGBT9 [61]. The entire open reading frame for the protein Reg1 and its derivative Reg1-P231L was expressed as a fusion to the Gal4 activation domain in the plasmid pACT2 (Clontech).

### RNAseq analysis

RNA samples were prepared from multiple independent yeast cultures grown on synthetic complete medium using the RNeasy Mini Kit (Qiagen). Sequencing libraries were prepared using the TruSeq Stranded mRNA library method (Illumina). RNA sequences were mapped to *S*. *cerevisiae* mRNA using the kallisto software package [36]. Each RNA sample yielded 40–50 million reads. mRNA abundance was expressed in transcripts per million mapped reads (tpm). Comparison of mRNA expression under different conditions utilized a student t-test to calculate a p values with a false discovery rate threshold of 0.01%.

### Fluorescence microscopy and quantification

Cells were grown to mid-log phase in synthetic complete medium containing 2% glucose and then incubated an additional 2 h after 0.2% glucose or 0.2% 2DG addition. Fluorescent images were acquired on a TiE2 inverted microscope (Nikon, Chiyoda, Tokyo, Japan) equipped with an Apo100x objective (NA 1.45) and captured with an Orca Flash 4.0 cMOS (Hammamatsu, Bridgewater, NJ) camera and NIS-Elements software (Nikon). To define vacuoles, cells were incubated with 250 uM Cell Tracker Blue CMAC dye (Life Technologies, Carlsbad, CA). All images were acquired using equivalent parameters and are presented as evenly adjusted images from Adobe Photoshop. For all figures an unsharp mask was applied with a threshold of 5 and a pixel radius of 5. Quantification of plasma membrane and vacuolar fluorescence intensities was assessed as described [10].

### Statistical significance

Unless otherwise stated, mean values represents the average for a minimum of three independent measurements, and the error bars represent 1 standard error. Statistical significance was determined using the Student t test for unpaired variables with equal variance. For statistical analysis of HXT3-GFP localization, populations were assessed for statistically significant differences using non-parametic Kruskal-Wallis statistical analyses with Dunn’s multiple comparisons post-hoc test using Prism software (GraphPad,La Jolla, CA). In all cases, p values are indicated as follows: * p<0.05; ** p<0.01; *** p<0.001.

## Supporting information

S1 Fig 2DG-resistant strains with aneuploidy. DNA sequence read depth across all sixteen chromosomes was plotted for the wild-type strain (MSY1333) and three 2DG-resistant strains. The median value for each chromosome is shown as a yellow bar.
(TIF)

S2 Fig Single point mutations are sufficient to confer 2DG resistance. Original isolates with candidate mutations located in the chromosomal loci (along with other variants of unknown significance) were compared to strains with gene deletions transformed with plasmids encoding wild type and mutant alleles. 2DG resistance was measured in quadruplicate by comparing growth in the absence of 2DG with growth in the presence of 0.1% 2DG. All strains bearing candidate mutations showed significant 2DG resistance (p<0.001) regardless of whether the mutation was encoded on the chromosome in the original isolate or reconstructed on a plasmid.
(TIF)

S3 Fig Growth properties and 2DG resistance in hexokinase deletion strains. A. Haploid yeast strains with the indicated genotypes were spotted onto agar plates at two different dilutions. Plates contained galactose (Gal), glycerol/ethanol (GE), glucose (Glu) or glucose plus 2DG. B. 2DG resistance assay was performed in triplicate at 0.1% 2DG in glucose media with wild type (WT) or strains lacking a single hexokinase gene. Mean values (±SD) are plotted with those statistically different from wild type indicated.
(TIF)

S4 Fig Kinetic analysis of Hxk1. Protein extracts were prepared from cells lacking all three hexokinase genes and transformed with a low-copy plasmid expressing Hxk1. Extracts were assayed for activity using a range of concentrations for the substrates glucose and 2-deoxyglucose. Production of NADPH was measured over time, and the rate of substrate phosphorylation was used to measure enzyme kinetic properties using a double reciprocal plot.
(TIF)

S5 Fig Kinetic analysis of Hxk2. Protein extracts were prepared from cells lacking all three hexokinase genes and transformed with a low-copy plasmid expressing Hxk2. Extracts were assayed for activity using a range of concentrations for the substrates glucose and 2-deoxyglucose. Production of NADPH was measured over time, and the rate of substrate phosphorylation was used to measure enzyme kinetic properties using a double reciprocal plot.
(TIF)

S6 Fig Kinetic analysis of Glk1. Protein extracts were prepared from cells lacking all three hexokinase genes and transformed with a low-copy plasmid expressing Glk1. Extracts were assayed for activity using a range of concentrations for the substrates glucose and 2-deoxyglucose. Production of NADPH was measured over time, and the rate of substrate phosphorylation was used to measure enzyme kinetic properties using a double reciprocal plot.
(TIF)

S7 Fig Protein-protein interactions between Reg1, Snf1 and Glc7. Two-hybrid interactions were measured as growth on synthetic complete medium with 2% glucose and the absence of histidine (-His). Yeast cells (*gal4Δ gal80Δ GAL7-HIS3*) were transformed with two-hybrid plasmids expressing the protein shown fused to the DNA binding domain or activation domain of Gal4. Homo-dimerization of herpes virus capsid protein 22a was used as a positive control when paired with itself and as a negative control when paired with Reg1 or Glc7.
(TIF)

S8 Fig Relative fitness of 2DG-resistant strains. Different yeast strains bearing various alleles of *HXK2*, *REG1*, *SNF1* and *GLC7*, as indicated, were grown competitively in synthetic complete media with 2% glucose (blue circles) or 2% glucose plus 0.1% 2DG (yellow squares). After growth, the representation of each genotype was determined based on different auxotrophic markers and the relative fitness index defined by Wiser and Lenski [32] is plotted. Positive values indicate a growth advantage to cells with the allele shown below in the numerator, while negative values indicate a growth advantage to cells with the allele shown below in the denominator. For the HXK2 alleles, we found that complete deletion of HXK2 exacted a very small fitness cost compared to wild type for cells growing on glucose and conferred a large fitness advantage to cells growing in the presence of 2DG. The missense alleles of HXK2 all showed a similar advantage in media with 2DG with very low cost on glucose. A very different fitness landscape is seen with REG1 alleles. Deletion of REG1 exacts a large fitness cost for cells growing on glucose (over 4-fold). Cells with reg1Δ or the reg1-P231L allele showed a large increase in fitness when challenged with 2DG compared to wild type REG1 cells. Interestingly, the reg1-P231L allele showed increased fitness compared to the reg1Δ allele in both glucose and glucose plus 2DG media. The loss of SNF1 came with a reduced fitness under both media conditions. The SNF1-G53R allele confers increased fitness compared to wild type when challenged with 2DG with almost no fitness cost to cells growing on glucose. The SNF1-G53R conferred an advantage to cells compared to the snf1Δ on both media. Finally, we compared the GLC7 wild-type allele with the glc7-Q48K allele. The glc7Δ could not be examined, since this strain is not viable. We found that the glc7-Q48K allele conferred a strong fitness advantage to cells challenged with 2DG, but it came with a detectable fitness cost when cells are grown on glucose. In summary, the relative fitness of the 2DG-resistant alleles could explain why missense alleles were recovered in the REG1, SNF1 and GLC7 genes but could not explain why we did not recover any nonsense alleles in HXK2.
(TIF)

S9 Fig RNAseq analysis of the transcriptional response to 2DG in resistant strains. Wild type and yeast strains bearing various alleles of *HXK2*, *REG1*, *SNF1* and *GLC7*, as indicated, were grown in triplicate cultures. RNA was collected after growth to mid-log and two hours after addition of 2DG to 0.1%. RNA abundance was quantified and is displayed here for 5917 mRNAs as the log2 ratio of the mean expression values (tpm) on the y-axis and the mean expression values under both conditions on the x-axis. Expression levels that show a statistically significant change using an adjusted p-value with a false-discovery rate threshold of 0.01 or lower are shown as colored circles. Those not meeting this threshold are shown as smaller black dots. Ribosomal protein genes (red circles), hexose transporter genes (yellow circles) and *DOG1* and *DOG2* genes (white diamonds) are indicated.
(TIF)

S1 Table mRNA abundance determined by RNAseq. mRNA abundance was determined by RNAseq using kallisto software and is reported here as tpm (transcripts per million mapped reads).
(XLSX)
