# Supplementary figures and images for: Spontaneous mutations that confer resistance to 2-deoxyglucose act through Hxk2 and Snf1 pathways to regulate gene expression and HXT endocytosis
Source: PLoS Genet. 2020 Jul 16;16(7):e1008484. doi: 10.1371/journal.pgen.1008484 (PMC7386655; doi:10.1371/journal.pgen.1008484)

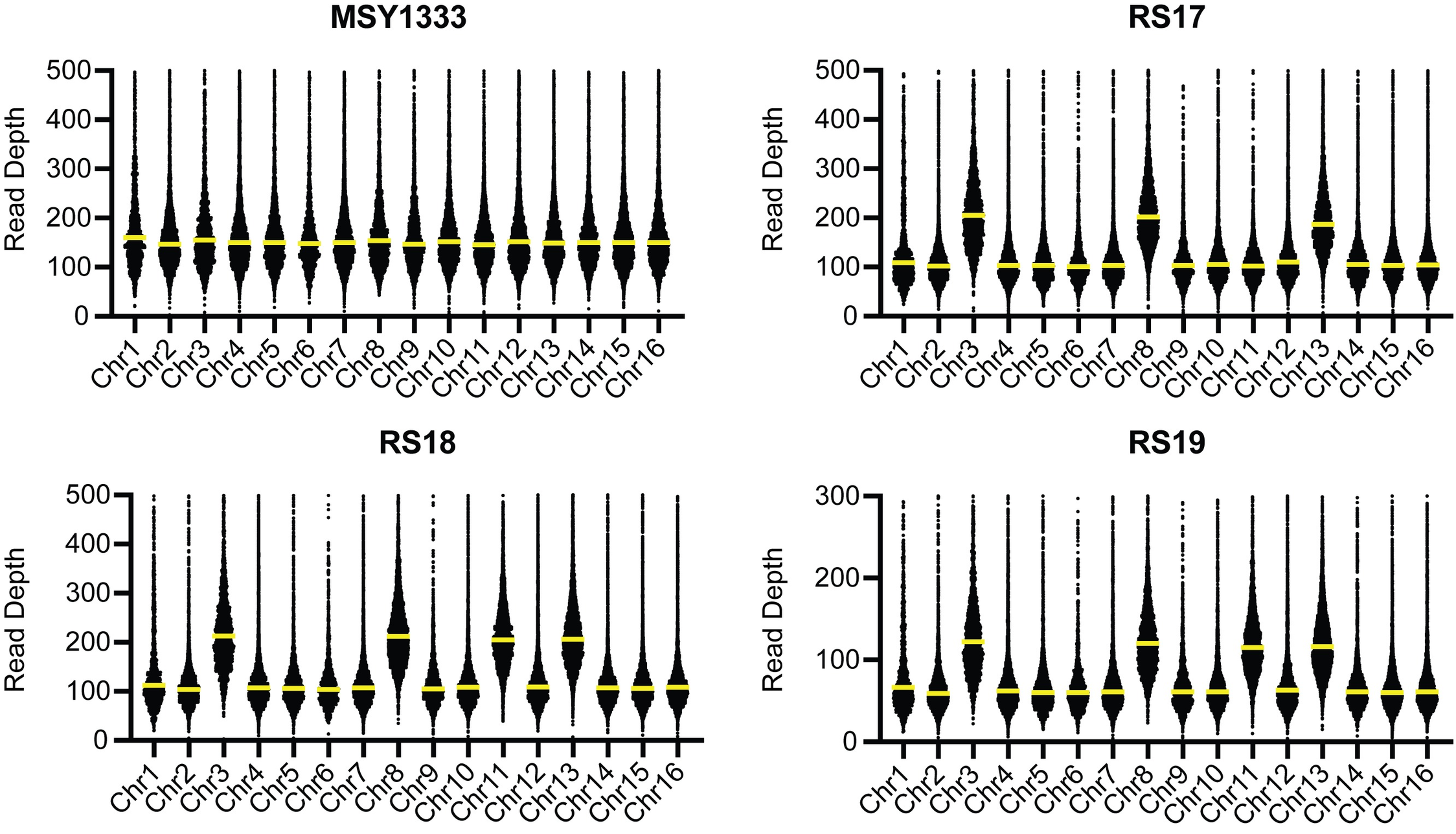

Supplement: S1 Fig — DNA sequence read depth across all sixteen chromosomes was plotted for the wild-type strain (MSY1333) and three 2DG-resistant strains. The median value for each chromosome is shown as a yellow bar. (TIF) [file pgen.1008484.s001.tif]

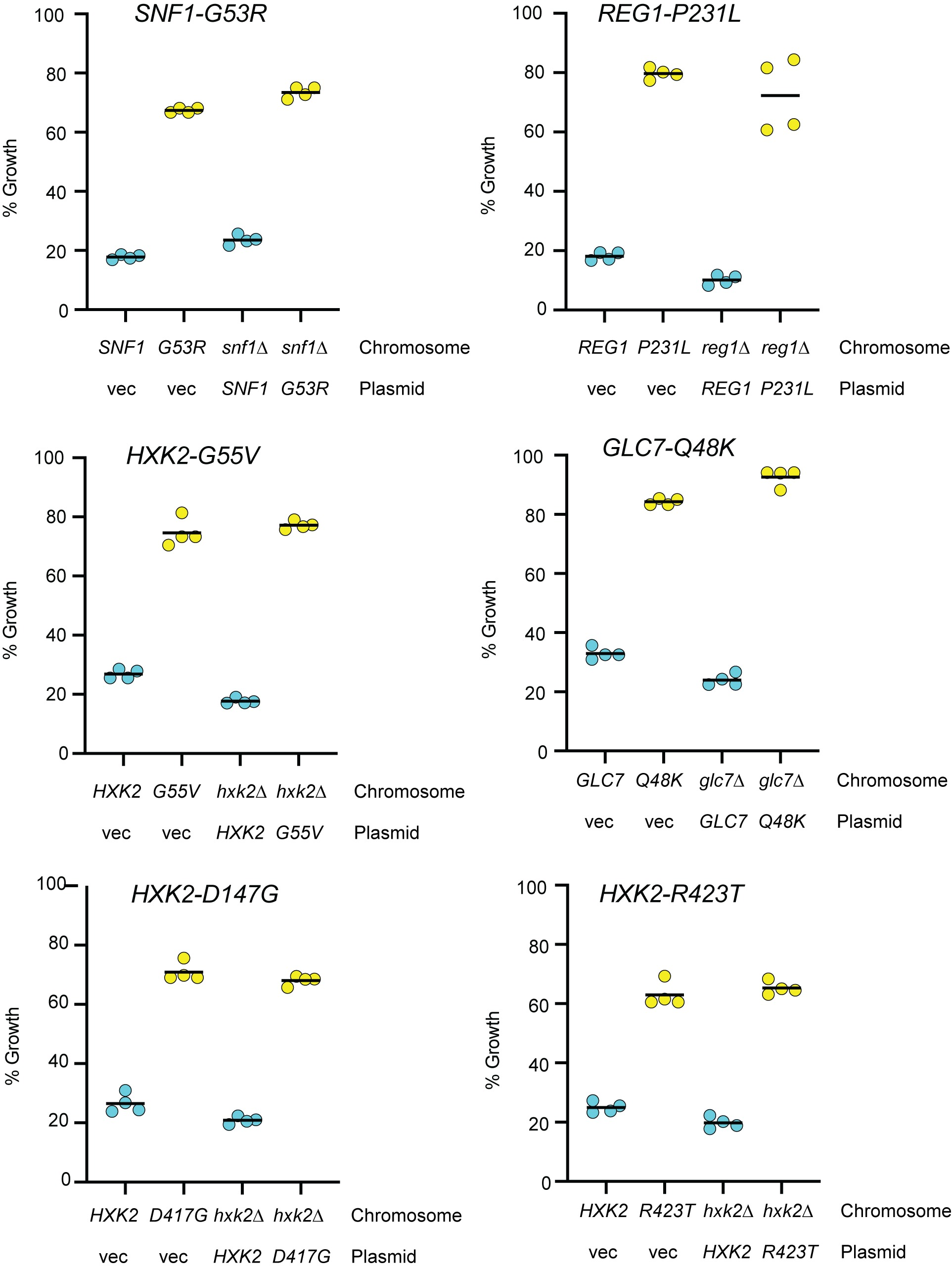

Supplement: S2 Fig — Original isolates with candidate mutations located in the chromosomal loci (along with other variants of unknown significance) were compared to strains with gene deletions transformed with plasmids encoding wild type and mutant alleles. 2DG resistance was measured in quadruplicate by comparing growth in the absence of 2DG with growth in the presence of 0.1% 2DG. All strains bearing candidate mutations showed significant 2DG resistance (p<0.001) regardless of whether the mutation was encoded on the chromosome in the original isolate or reconstructed on a plasmid. (TIF) [file pgen.1008484.s002.tif]

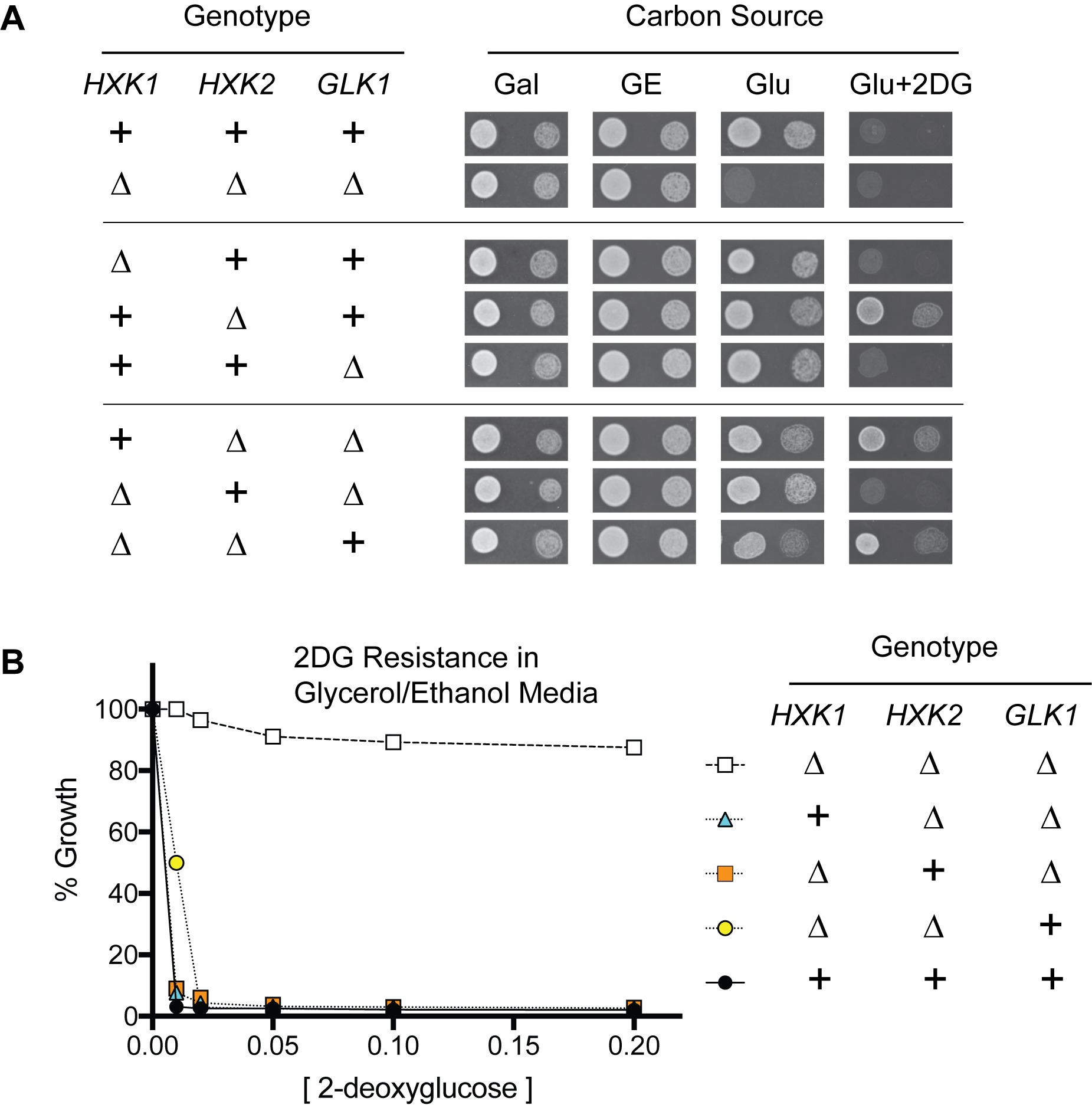

Supplement: S3 Fig — A. Haploid yeast strains with the indicated genotypes were spotted onto agar plates at two different dilutions. Plates contained galactose (Gal), glycerol/ethanol (GE), glucose (Glu) or glucose plus 2DG. B. 2DG resistance assay was performed in triplicate at 0.1% 2DG in glucose media with wild type (WT) or strains lacking a single hexokinase gene. Mean values (±SD) are plotted with those statistically different from wild type indicated. (TIF) [file pgen.1008484.s003.tif]

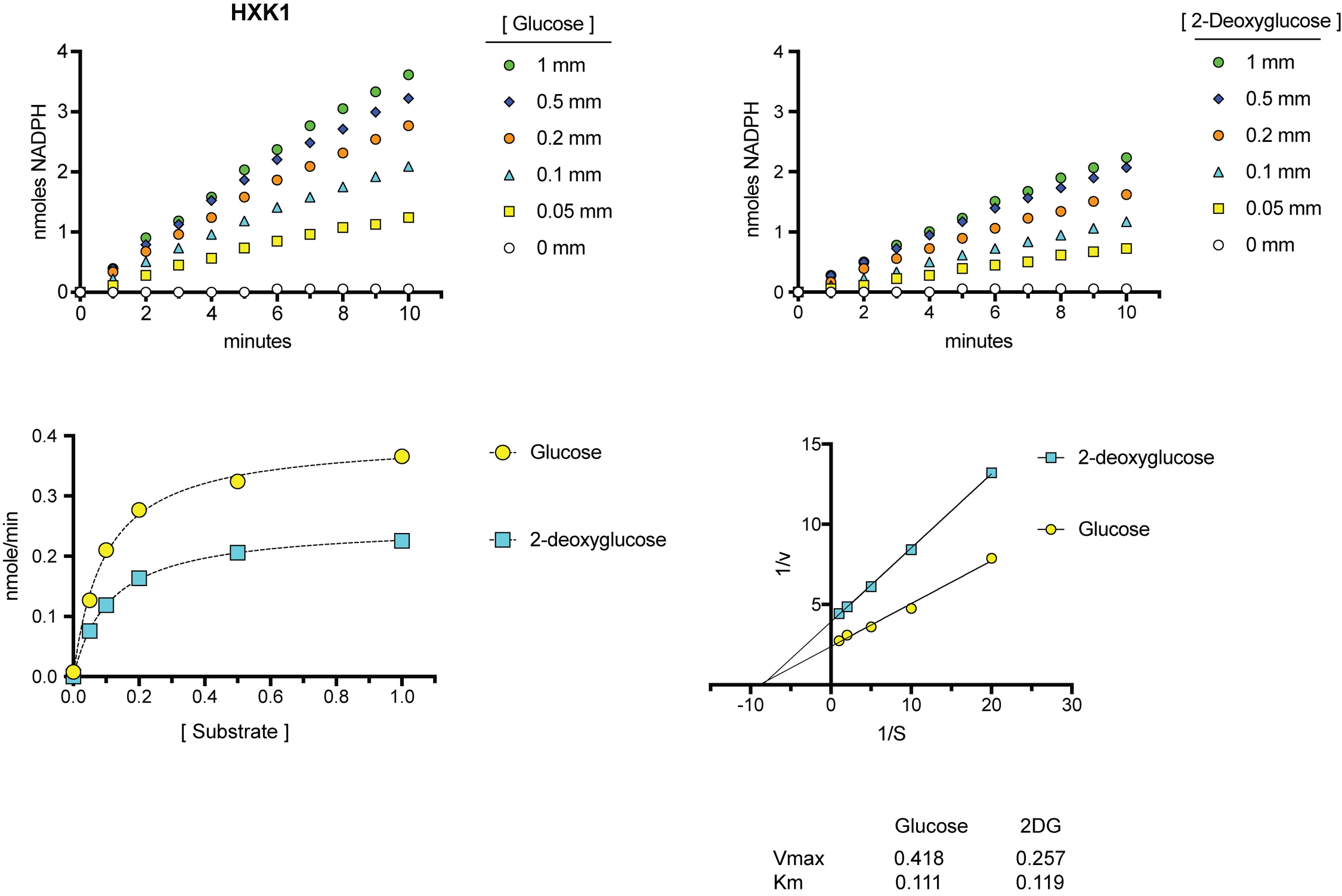

Supplement: S4 Fig — Protein extracts were prepared from cells lacking all three hexokinase genes and transformed with a low-copy plasmid expressing Hxk1. Extracts were assayed for activity using a range of concentrations for the substrates glucose and 2-deoxyglucose. Production of NADPH was measured over time, and the rate of substrate phosphorylation was used to measure enzyme kinetic properties using a double reciprocal plot. (TIF) [file pgen.1008484.s004.tif]

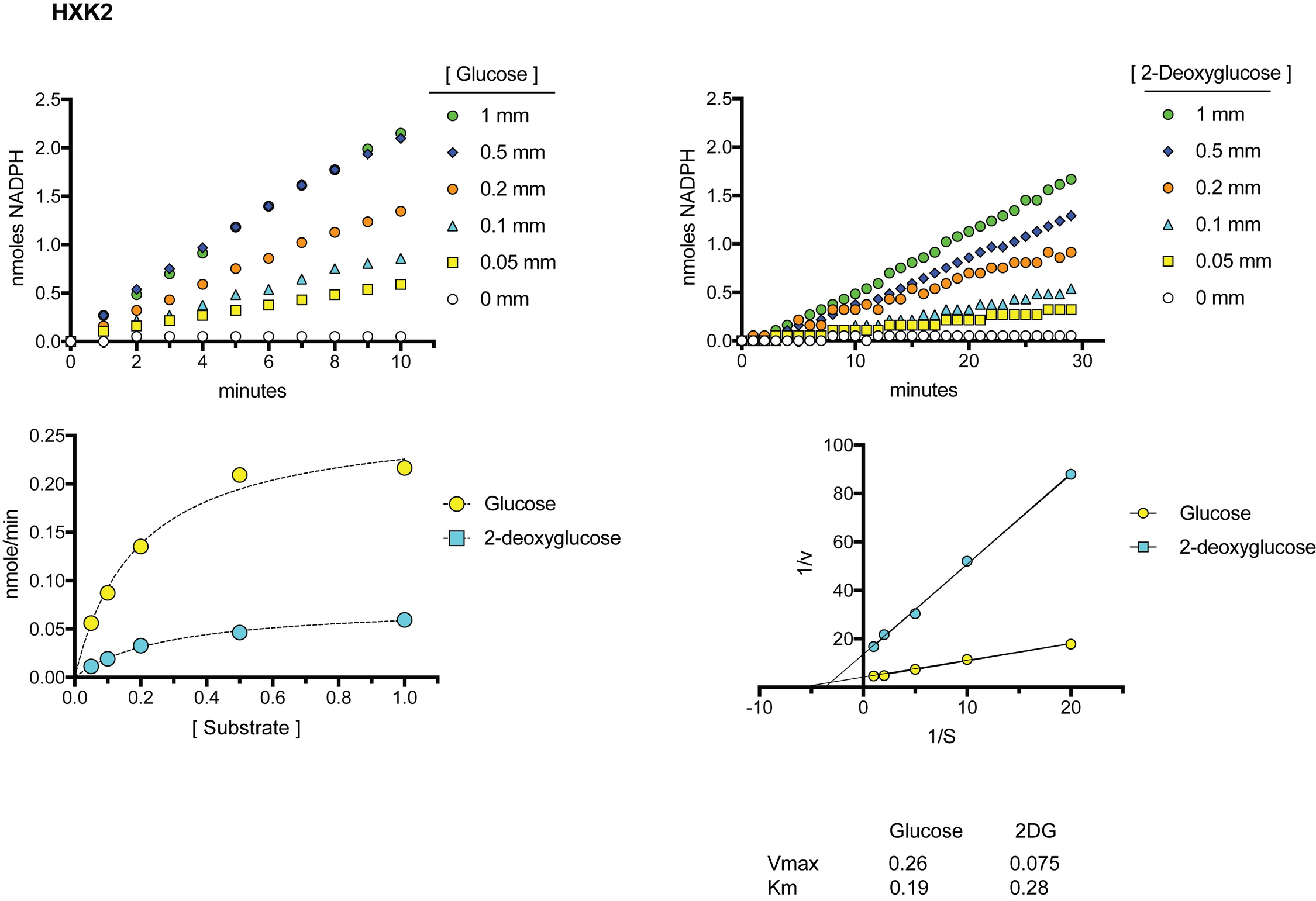

Supplement: S5 Fig — Protein extracts were prepared from cells lacking all three hexokinase genes and transformed with a low-copy plasmid expressing Hxk2. Extracts were assayed for activity using a range of concentrations for the substrates glucose and 2-deoxyglucose. Production of NADPH was measured over time, and the rate of substrate phosphorylation was used to measure enzyme kinetic properties using a double reciprocal plot. (TIF) [file pgen.1008484.s005.tif]

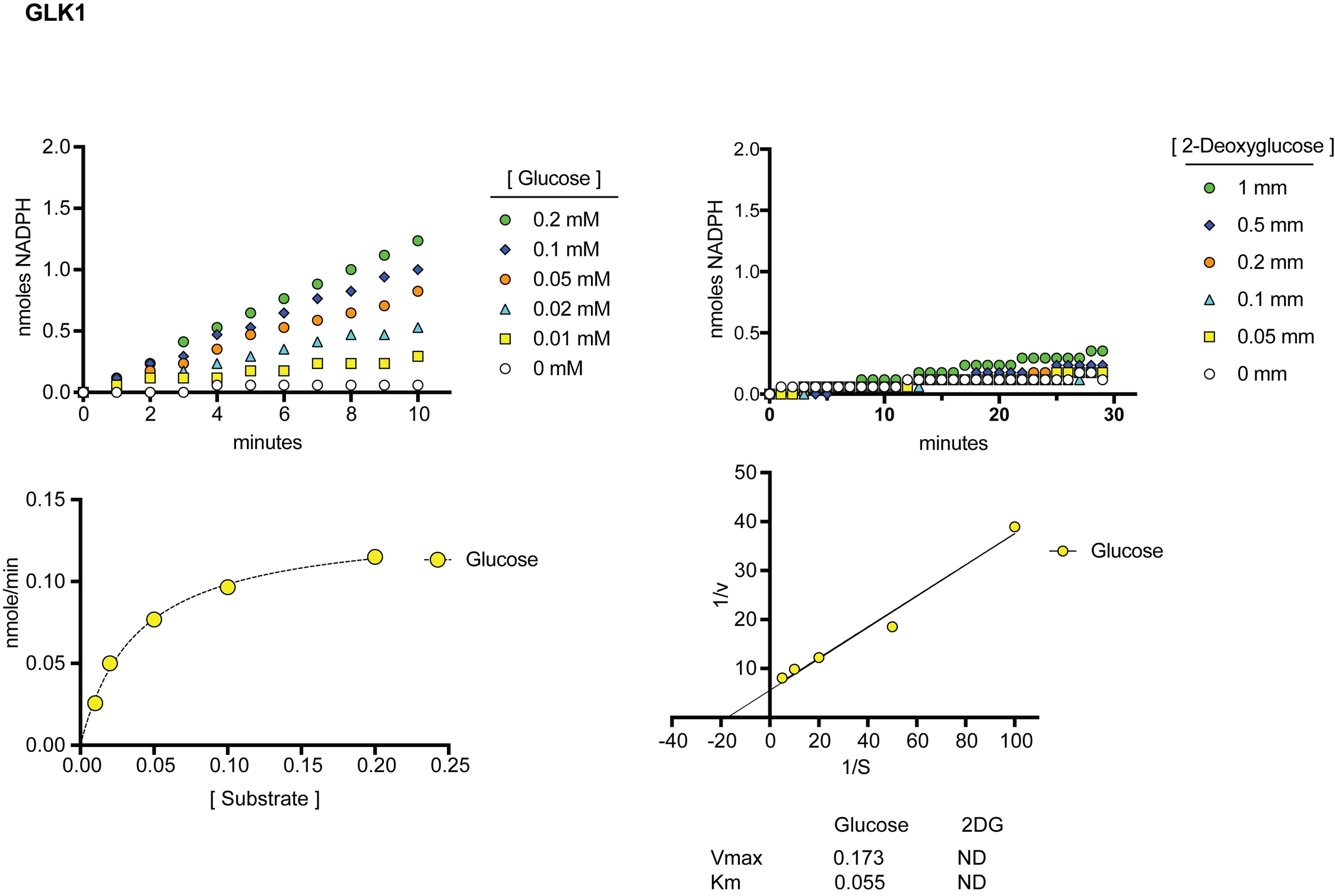

Supplement: S6 Fig — Protein extracts were prepared from cells lacking all three hexokinase genes and transformed with a low-copy plasmid expressing Glk1. Extracts were assayed for activity using a range of concentrations for the substrates glucose and 2-deoxyglucose. Production of NADPH was measured over time, and the rate of substrate phosphorylation was used to measure enzyme kinetic properties using a double reciprocal plot. (TIF) [file pgen.1008484.s006.tif]

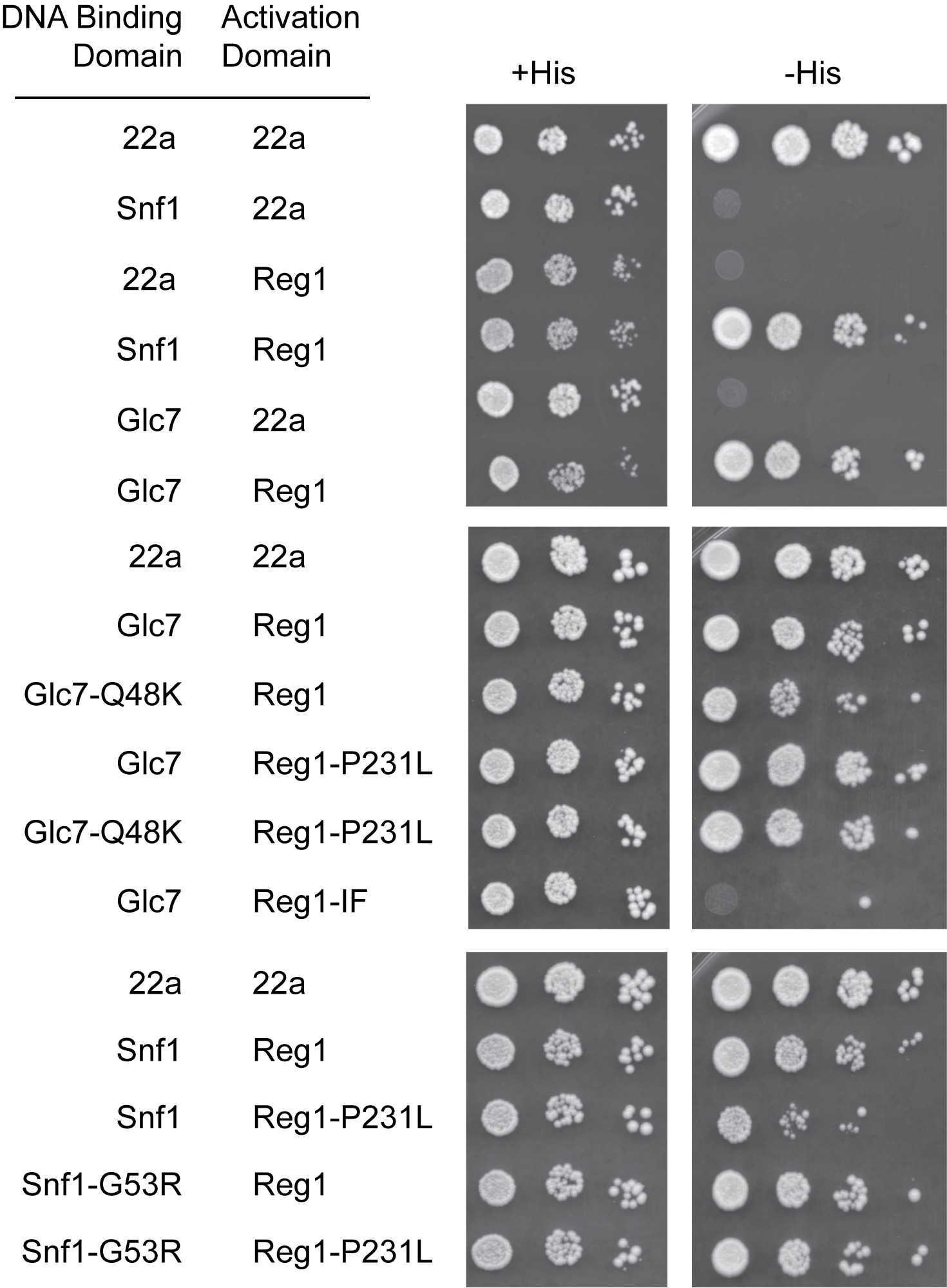

Supplement: S7 Fig — Two-hybrid interactions were measured as growth on synthetic complete medium with 2% glucose and the absence of histidine (-His). Yeast cells (gal4Δ gal80Δ GAL7-HIS3) were transformed with two-hybrid plasmids expressing the protein shown fused to the DNA binding domain or activation domain of Gal4. Homo-dimerization of herpes virus capsid protein 22a was used as a positive control when paired with itself and as a negative control when paired with Reg1 or Glc7. (TIF) [file pgen.1008484.s007.tif]

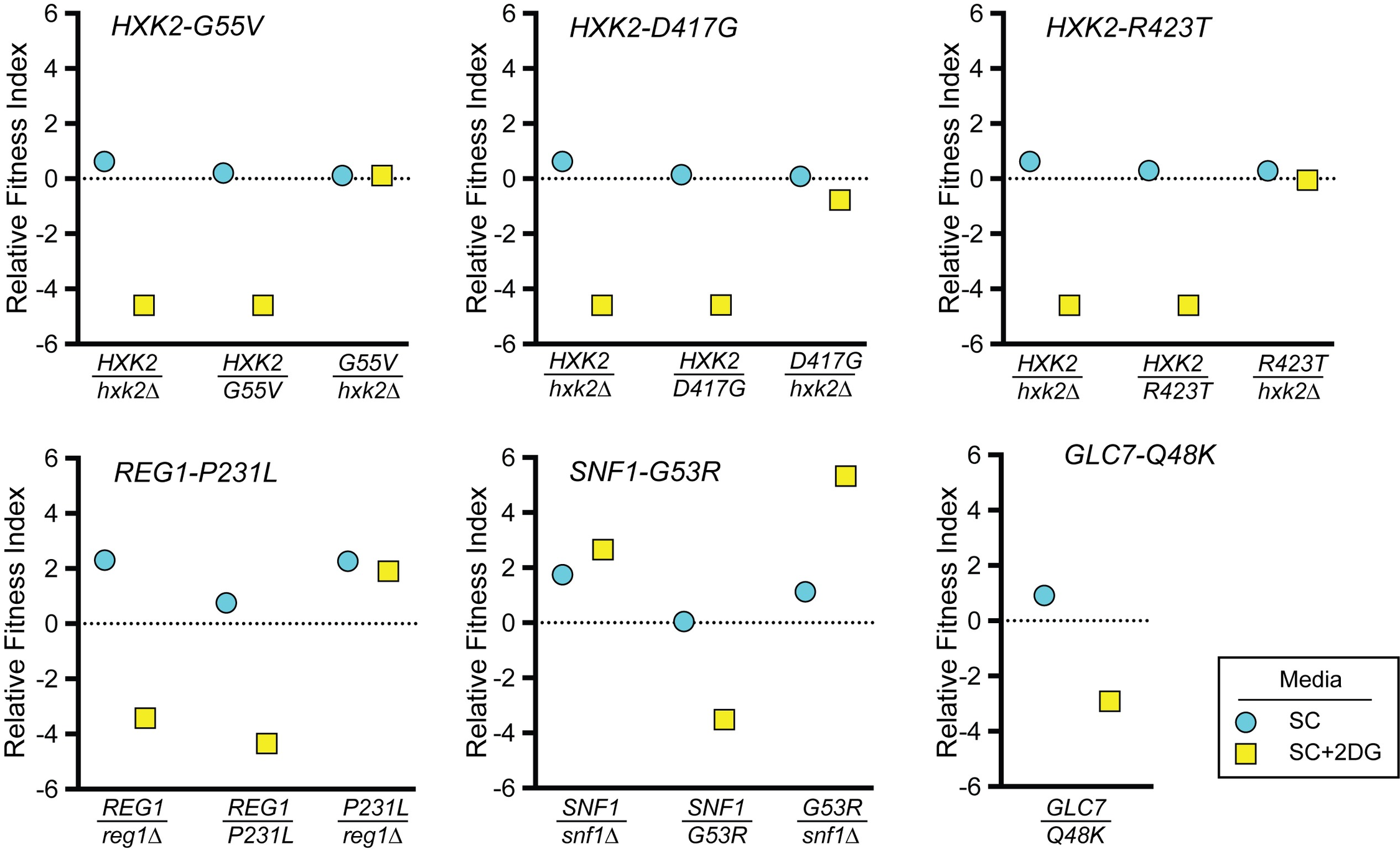

Supplement: S8 Fig — Different yeast strains bearing various alleles of HXK2, REG1, SNF1 and GLC7, as indicated, were grown competitively in synthetic complete media with 2% glucose (blue circles) or 2% glucose plus 0.1% 2DG (yellow squares). After growth, the representation of each genotype was determined based on different auxotrophic markers and the relative fitness index defined by Wiser and Lenski [32] is plotted. Positive values indicate a growth advantage to cells with the allele shown below in the numerator, while negative values indicate a growth advantage to cells with the allele shown below in the denominator. For the HXK2 alleles, we found that complete deletion of HXK2 exacted a very small fitness cost compared to wild type for cells growing on glucose and conferred a large fitness advantage to cells growing in the presence of 2DG. The missense alleles of HXK2 all showed a similar advantage in media with 2DG with very low cost on glucose. A very different fitness landscape is seen with REG1 alleles. Deletion of REG1 exacts a large fitness cost for cells growing on glucose (over 4-fold). Cells with reg1Δ or the reg1-P231L allele showed a large increase in fitness when challenged with 2DG compared to wild type REG1 cells. Interestingly, the reg1-P231L allele showed increased fitness compared to the reg1Δ allele in both glucose and glucose plus 2DG media. The loss of SNF1 came with a reduced fitness under both media conditions. The SNF1-G53R allele confers increased fitness compared to wild type when challenged with 2DG with almost no fitness cost to cells growing on glucose. The SNF1-G53R conferred an advantage to cells compared to the snf1Δ on both media. Finally, we compared the GLC7 wild-type allele with the glc7-Q48K allele. The glc7Δ could not be examined, since this strain is not viable. We found that the glc7-Q48K allele conferred a strong fitness advantage to cells challenged with 2DG, but it came with a detectable fitness cost when cells are grown on glucose. In summ [file pgen.1008484.s008.tif]

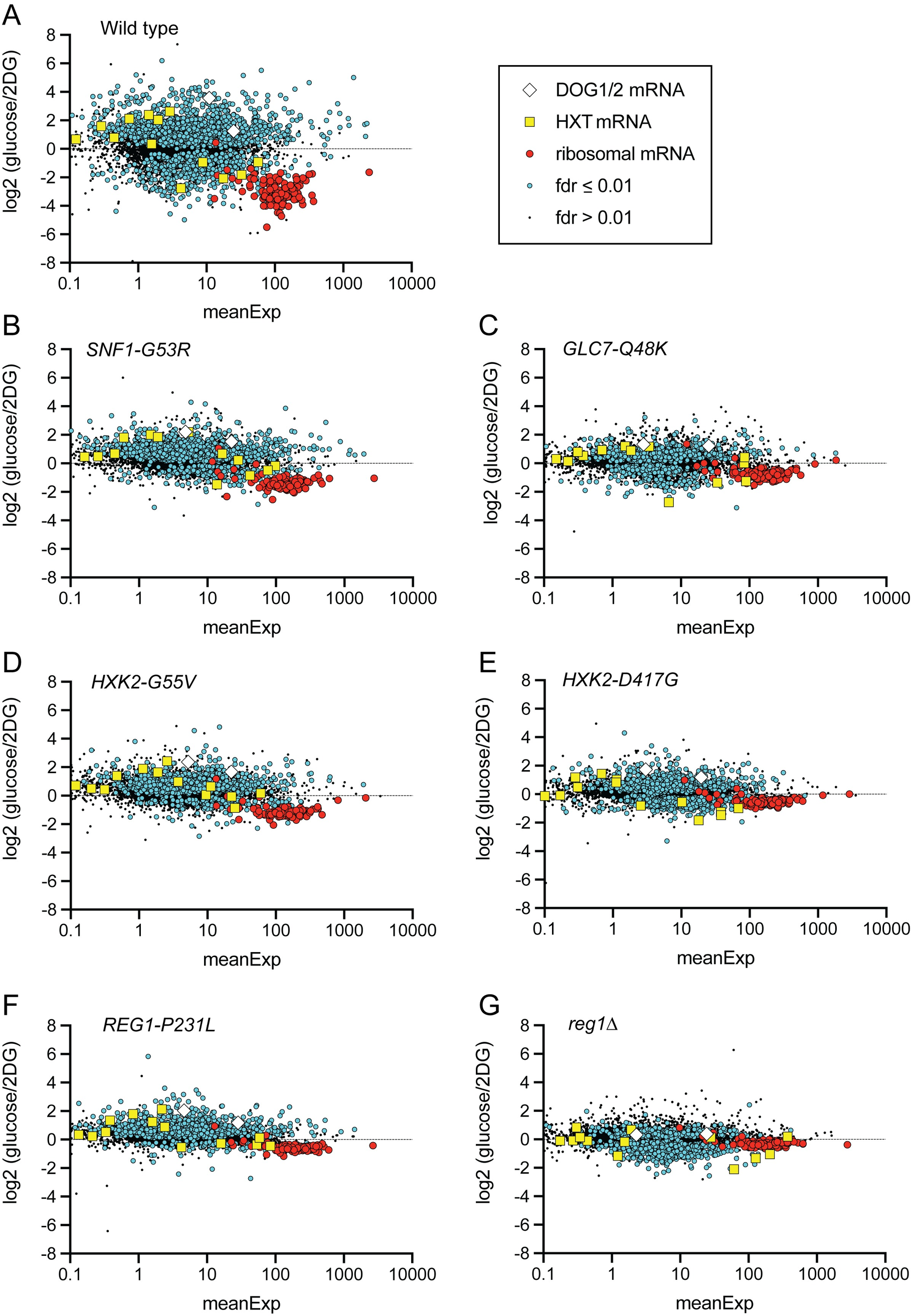

Supplement: S9 Fig — Wild type and yeast strains bearing various alleles of HXK2, REG1, SNF1 and GLC7, as indicated, were grown in triplicate cultures. RNA was collected after growth to mid-log and two hours after addition of 2DG to 0.1%. RNA abundance was quantified and is displayed here for 5917 mRNAs as the log2 ratio of the mean expression values (tpm) on the y-axis and the mean expression values under both conditions on the x-axis. Expression levels that show a statistically significant change using an adjusted p-value with a false-discovery rate threshold of 0.01 or lower are shown as colored circles. Those not meeting this threshold are shown as smaller black dots. Ribosomal protein genes (red circles), hexose transporter genes (yellow circles) and DOG1 and DOG2 genes (white diamonds) are indicated. (TIF) [file pgen.1008484.s009.tif]
